# The impact of VPS35 D620N mutation on alternative autophagy and its reversal by estrogen in Parkinson's disease

**DOI:** 10.1007/s00018-024-05123-4

**Published:** 2024-02-27

**Authors:** Tomotaka Shiraishi, Keiko Bono, Hiromi Hiraki, Yoko Manome, Hisayoshi Oka, Yasuyuki Iguchi, Hirotaka James Okano

**Affiliations:** 1 Division of Regenerative Medicine, The Jikei University School of Medicine, 3‑25‑8 Nishi‑Shinbashi, Minato‑ku, Tokyo, 1058461 Japan; 2 Department of Neurology, The Jikei University School of Medicine, 3‑25‑8 Nishi‑Shinbashi, Minato‑ku, Tokyo, 105‑8461 Japan

**Keywords:** Parkinson’s disease, VPS35, Retromer, Alternative autophagy, Estrogen, Rab9

## Abstract

VPS35 plays a key role in neurodegenerative processes in Alzheimer’s disease and Parkinson’s disease (PD). Many genetic studies have shown a close relationship between autophagy and PD pathophysiology, and specifically, the PD-causing D620N mutation in VPS35 has been shown to impair autophagy. However, the molecular mechanisms underlying neuronal cell death and impaired autophagy in PD are debated. Notably, increasing evidence suggests that Rab9-dependent “alternative” autophagy, which is driven by a different molecular mechanism that driving ATG5-dependent “conventional” autophagy, also contributes to neurodegenerative process. In this study, we investigated the relationship between alternative autophagy and VPS35 D620N mutant-related PD pathogenesis. We isolated iPSCs from the blood mononuclear cell population of two PD patients carrying the VPS35 D620N mutant. In addition, we used CRISPR-Cas9 to generate SH-SY5Y cells carrying the D620N variant of VPS35. We first revealed that the number of autophagic vacuoles was significantly decreased in ATG5-knockout Mouse Embryonic Fibroblast or ATG5-knockdown patient-derived dopaminergic neurons carrying the VPS35 D620N mutant compared with that of the wild type VPS35 control cells. Furthermore, estrogen, which activates alternative autophagy pathways, increased the number of autophagic vacuoles in ATG5-knockdown VPS35 D620N mutant dopaminergic neurons. Estrogen induces Rab9 phosphorylation, mediated through Ulk1 phosphorylation, ultimately regulating alternative autophagy. Moreover, estrogen reduced the apoptosis rate of VPS35 D620N neurons, and this effect of estrogen was diminished under alternative autophagy knockdown conditions. In conclusion, alternative autophagy might be important for maintaining neuronal homeostasis and may be associated with the neuroprotective effect of estrogen in PD with VPS35 D620N.

**Supplementary Information:**

The online version contains supplementary material available at 10.1007/s00018-024-05123-4.

## Background

Parkinson’s disease (PD) is the second most common neurodegenerative disorder after Alzheimer’s disease (AD), and it is characterized by the loss of dopaminergic neurons in the substantia nigra pars compacta, causing debilitating motor deficits. Pathologically, PD is a synucleinopathy, a neurodegenerative disease characterized by the abnormal accumulation of alpha-synuclein (α-syn) [1]. Although most PD cases are idiopathic, monogenic forms of the disease are evident in up to 10% of patients. Vacuolar protein sorting 35 (VPS35) has been reported to be a pathogenic gene in autosomal dominant PD. A single missense mutation, c.1858G > A (p.D620N), was initially shown to segregate with PD in Swiss and Austrian families [2, 3]. In addition, a previous study has shown that the VPS35 expression levels were reduced in the substantia nigra of sporadic PD patients. Intriguingly, reduced protein levels of VPS35 have also been found in the entorhinal cortex of AD patients [4], leading to an increased focus on the role played by VPS35 in the pathophysiology of neurodegenerative diseases.

The VPS35 protein is a functional subunit of the trimer complex (VPS26-VPS29-VPS35) referred to as the retromer complex [5]. First identified in yeast, the retromer complex has been shown to be critical for retrograde transport of various proteins (“cargo”) from the endosomal network to either the trans-Golgi network (TGN) or the plasma membrane by promoting the formation of endosomal membrane tubules in which the cargo proteins are sorted [5]. However, it remains to be determined how the retrograde transport function of the retromer complex is associated with the pathogenesis of PD with the VPS35 D620N mutation.

In terms of the pathogenesis of neurodegenerative diseases, autophagy plays a key role in degrading defective proteins, including α-syn, and is essential for neuronal homeostasis, as many genetic studies have shown a close relationship between autophagy and PD pathophysiology [6]. Given that the cation-independent mannose 6-phosphate receptor (CI-MPR) is one of the best-characterized retromer cargoes and participates in the delivery of lysosomal enzymes to lysosomes [7], alternations in CI-MPR trafficking caused by the D620N mutation can have a significant influence on autophagy [8]. Furthermore, Zavodszky et al. revealed that the D620N mutation affects the localization and trafficking of ATG9A [9], which is necessary for proper autophagy induction, and other studies have also reported that the PD-causing D620N mutation in VPS35 impairs autophagy [10, 11]. However, the molecular mechanisms underlying neuronal cell death and autophagy impairment have not been established. In addition, while a number of studies have been performed with molecules targeting autophagy in vitro and in vivo, and even clinical studies have been conducted, none of these studies have led to the approval of the tested molecules for the treatment of PD [12].

Moreover, accumulating evidence has suggested that “alternative” autophagy, which involves a molecular mechanism distinct from that involved in “conventional” autophagy, contributes to the neurodegenerative process [13, 14]. In alternative autophagy, autophagosomes are formed at the trans-Golgi membrane, whereas the endoplasmic reticulum and mitochondria are crucial for initiating conventional autophagy. Rab9 is the unique core machinery in alternative autophagy; in contrast, ATG5, ATG7, ATG9, and LC3 are crucial for conventional autophagy [15]. In this study, we found that Rab9-dependent alternative autophagy was impaired by the D620N mutation of VPS35 and that the activation of alternative autophagy decreased the vulnerability of patient-derived VPS35 D620N mutant neurons.

## Materials and methods

### Generation of iPSCs and neuronal induction

We used 201B7 cells obtained from the RIKEN BioResource Centre as Control_1 cells. All PD induced pluripotent stem cells (iPSCs) and Control_2 iPSCs were generated from human peripheral blood mononuclear cells using episomal vectors according to a protocol established at the Centre for iPSC Cell Research and Application (Kyoto University, Japan) and previously described [16]. All iPSC lines were induced to differentiate into dopaminergic neurons with a protocol established by Reinhardt et al. [17]. For reactive oxygen species (ROS) and cleaved caspase-3 (CC3) measurements and a Cyto-ID autophagy assessment, neurons were cultivated in neuronal medium without B27 or ascorbic acid (that is, without antioxidant supplementation) 4 h prior to the experiment [11]. In meeting ethics guidelines, approval for the generation of patient-derived iPSCs was granted after obtaining informed consent of the patients.

### Generation of VPS35 D620N SH-SY5Y cells

The D620N mutation in VPS35 was obtained by Clustered Regularly Interspaced Short Palindromic Repeats (CRISPR)-Cas9–mediated genome editing [18, 19] in the SH-SY5Y neuroblastoma cell line. We followed the methods outlined by Ma et al. [19], in which a single guide RNA (sgRNA) and a single-stranded oligodeoxynucleotide sequence were designed to facilitate homology-directed repair of the endogenous locus. This design included the substitution of five nucleotides: a G > A nucleotide substitution leading to the D620N mutation of VPS35, and four synonymous substitutions creating a novel EcoRI restriction site. This site was introduced to prevent repetitive cutting of Cas9 by the repair template, thereby avoiding unintended cleavage. The sgRNA sequence was cloned into the px330.puro plasmid (a gift from Sandra Martha Gomes Dias, Addgene plasmid #110403). Subsequently, the px330 plasmid and the single-stranded oligonucleotides were transfected into the SH-SY5Y cells using NucleofectorII (Amaxa) following the manufacturer’s protocol (Lonza). Following transfection, antibiotic selection with puromycin was initiated 48 h later. Surviving cells were then subjected to single-cell cloning and screened for the presence of the D620N mutation using the EcoRI restriction enzyme. In parallel, a batch of cells was mock-electroporated and served as the wild type control in subsequent experiments. Finally, we sequenced the three predicted off-target genomic regions within coding regions of genes POU6F1, ZNF318 and KY [19], but found no off-target edits. Primer and template sequences are the same as the previous study [19].

### Cell culture

HeLa cells were grown in DMEM supplemented with 10% fetal bovine serum (FBS) and 1% penicillin–streptomycin with 5% CO_2_ in a humid incubator at 37 °C. SH-SY5Y cells were grown in DMEM/F-12 supplemented with 10% FBS, Non-Essential Amino Acids Solution, and 1% penicillin–streptomycin. Atg5^-/-^ mouse embryonic fibroblasts (MEFs) were gifted by Dr. Noboru Mizushima. To differentiate SH-SY5Y cells, cells were treated with 10 μM all trans retinoic acid (Sigma–Aldrich) for seven days. To induce autophagy, HeLa cells and MEFs were cultured with Hank’s balanced salt solution (HBSS; Thermo Fisher Scientific) for 4 h.

### Plasmid and short interfering RNA (siRNA) transfection

The VPS35 open reading frame (ORF) was subcloned into a pcDNA 3.1(+) vector, and the D620N mutation was generated using a PrimeSTAR Mutagenesis basal kit (Takara) with primers designed according to kit manufacturer’s instructions. The wild-type (WT) human Rab9 plasmid construct was obtained from Addgene (Plasmid #12663 [20]). Syntaxin7 and GS15 ORF was subcloned from HeLa cell cDNA. Transfection was performed in OptiMEM (Gibco) using Lipofectamine 3000 (Thermo Fisher Scientific) according to the manufacturer’s instructions. For siRNA-based knockdown, cells were transfected with RNAiMAX (Thermo Fisher Scientific) according to the manufacturer’s instructions. The following oligonucleotides were used: VPS35, 5′- GCCUUCAGAGGAUGUUGUAUCUUUAtt-3′ [8]; Rab9_1, 5′-GUUUGAUACCCAGCUCUUCtt-3′ [21]; Rab9_2, 5′- GAACAGAUAUGUAACUAAUtt-3′; ATG5, 5′-UAUCUCAUCCUGAUAUAGCgt-3′, ATG7, 5′-GGAACACUGUAUAACACCAtt-3′ [22]; WASH1, 5′-ACUACUUCUAUGUGCCAGAtt-3′ [23]; Wipi3, 5′-GGGAUGACCUGAAGAAGAAtt-3′; and EGFP (control), 5′-CAGCACGACUUCUUCAAGUtt-3′; a scrambled siRNA was also used (Cat# SR30004, OriGene Technologies Inc.). Rab9_1 was used for HeLa cells and a mixture of Rab9_1 and Rab9_2 was used for SH-SY5Y cells. For immunocytochemical analyses, cells were fixed 24–48 h after transfection. HeLa cells and Atg5^-/-^ MEFs stably expressing Flag-VPS35 constructs were generated and cultured in 400 µg/mL G418 (Enzo). Antibiotic selection was initiated 48 h after transfection and was continued for at least three weeks. For time‑lapse fluorescence microscopy, 24 h after transfection, cells were imaged at 37 °C in a stage incubator (Carl Zeiss), and time-lapse fluorescence images were acquired with a confocal laser microscope (LSM880, Carl Zeiss).

### Estrogen treatment

Cells were treated with 10 nmol/L 17β-estradiol for 24 h prior to analyses. Because phenol red possesses estrogenic properties, the cells were incubated with phenol red-free medium in experiments involving estrogen treatment [24].

### Immunocytochemical analysis

The cells were fixed with 4% paraformaldehyde for 5 min at room temperature (RT). After permeabilization with 0.3% Triton X-100 for 10 min and blocking with 5% bovine serum albumin (BSA) for 90 min, the cells were incubated overnight with primary antibodies at 4 °C. Primary antibodies against the following proteins were used for these analyses: MAP2 (chicken, Abcam), TH (mouse, Millipore), cleaved caspase-3 (rabbit, Cell Signaling), LAMP1 (mouse, Santa Cruz), Rab9 (rabbit, Abcam), and α-synuclein (rabbit, Cell Signaling), GPR30 (rabbit, #107748, GeneTex), WASH1 (mouse, SAB4200552, Sigma-Aldrich). The following day, the cells were washed with PBS and incubated with secondary antibodies for 60 min at RT. The secondary antibodies consisted of goat or donkey antibodies conjugated to Alexa 488, 546, 633, or 647 (Invitrogen). Nuclear staining was performed with a Hoechst solution (Invitrogen) containing the secondary antibodies.

### Image analysis

All image analyses were performed with ImageJ software (National Institute of Health, USA). The channels were split, and the background was subtracted with a rolling ball filter in a radius = 50 pixels. Median filters were applied (radius = 2 pixels). Colocalization analysis of Rab9 and LAMP1 was performed by calculating Pearson’s coefficients. To quantify the autophagic vacuoles in HeLa cells by Cyto-ID assay, we applied the fixed threshold in the “analyze particles” plugin. A circularity from 0.2 to 1.0 was fixed as a parameter for particle identification.

For time-lapse analysis of VPS35-Venus and mCherry-Rab9 in HeLa cells, we followed the methods described by Wong et al. [25]. All interactions analyzed for contact duration were established before the beginning of the video. The duration of each contact was quantified as the time before dissociation (VPS35 and Rab9 detaching from each other) at 3-s intervals over a 3-min period. Any contacts that lasted throughout the entire 3-min video were categorized as 180 s in the bar graphs and as > 180 s in the histograms. For fusion, fission, and tubule analyses with EGFP-Rab9 vesicles in HeLa stable cell lines expressing VPS35 WT or D620N, images were taken at 2-s intervals over a 3-min period. Fusion was defined as two Rab9 vesicles merging for at least 6 s, and fission was defined as one Rab9-carrying vesicle dividing into two vesicles and maintaining separation for at least for 6 s. In these time-lapse analyses, we excluded some mCherry-Rab9 or EGFP-Rab9 vesicles that had aggregated with each other or that had disappeared from the field during filming of the 3-min video. For the tubule formation analysis of EGFP-Rab9 and mCherry-Rab10, images were obtained at 3-s intervals.

### Western blot analysis

Protein extraction from cultured cells was performed with a mixture of Complete Mini (Roche), a phosphatase inhibitor cocktail (Nacalai Tesque), and Tissue Extraction Reagent I (Invitrogen) added to each culture well. After clear protein dissociation, centrifugation was performed at 15,000 rpm for 5 min. Samples were diluted with equal volumes of Laemmli sample buffer (Bio–Rad) and 5% 2-mercaptoethanol, incubated for 5 min at 95 °C and precipitated with a PAGE Clean Up kit (Nacalai Tesque). Protein samples were separated by SDS–PAGE and transferred to polyvinylidene difluoride (PVDF) membranes (Millipore). For the analysis of Rab9 phosphorylation, 100 μM Phos-tag AAL-107 (Wako) and 10 mM MnCl_2_ (Nacalai Tesque) were added to the acrylamide solutions. For the elimination of manganese ions, gels were soaked with transfer buffer containing 1 mM EDTA for 20 min and washed with transfer buffer without EDTA for 10 min. The membranes were blocked with PVDF Blocking Reagent for Can Get Signal (TOYOBO) for 16 h at 4 °C and incubated in primary antibody solution for 1 h at RT. Then, the membranes were incubated with secondary antibodies for 1 h at RT. Signals were detected with chemiluminescence horseradish peroxidase (HRP) Substrate (Takara Bio) and a WSE-6100 LuminoGraphI (Atto). A semiquantitative analysis of the signals was performed with ImageJ software. Primary antibodies against the following proteins were used for these analyses: Ulk1 (rabbit, #5869, Cell Signaling), Phospho-ULK1 (Ser555) (rabbit, #8054, Cell Signaling), Estrogen receptor *α* (rabbit, #127978, GeneTex), Estrogen receptor *β* (rabbit, #134663, GeneTex).

### Co-immunoprecipitation

To study protein interactions, co-immunoprecipitation (co-IP) assays were performed. HEK293T cells were transfected with mCherry-Rab9 and Flag-VPS35 using Lipofectamine 3000. The cells were lysed in immunoprecipitation buffer (50 mM Tris–HCl, 150 mM NaCl, and 1% NP40). Cell lysate was then incubated with anti-DDDDK-tag antibody (MBL) at 4 °C on a rotor for 2 h, and then incubated with Protein G Mag Sepharose™ beads (Cytiva) at 4 °C on a rotor for 2 h. The gel beads were washed three times with IP buffer, resuspended in 2X Laemmli Sample Buffer, separated by SDS-PAGE and analyzed by immunoblotting with specified antibodies.

### Flow cytometry

For Cyto-ID and CellROX fluorescence measurement, MEFs and SH-SY5Y cells were stained with Cyto-ID and CellROX reagent, respectively, according to the manufacturer’s instructions. The cells were dissociated using Trypsin for 3 min at 37 °C. The resulting cell suspension was analyzed with a MACSQuant Analyzer 10 flow cytometer (Miltenyi Biotec). Due to the nonparametric distribution of some samples, median fluorescence values were extracted for Cyto-ID and CellROX staining. Median fluorescence of the unstained cells was subtracted to account for autofluorescence.

### Statistical analysis

Statistical analysis was performed by R (version 3.6.1) software, utilizing either the parametric Student’s t-test or the nonparametric Mann–Whitney *U* test, with p < 0.05 considered significant. Significant differences among the three or more groups were determined by two-tailed multiple *t* tests with Bonferroni correction following the Kruskal–Wallis test for continuous variables.

## Results

### Impairment of Rab9-dependent alternative autophagy in VPS35 D620N cells

**Fig. 1:**
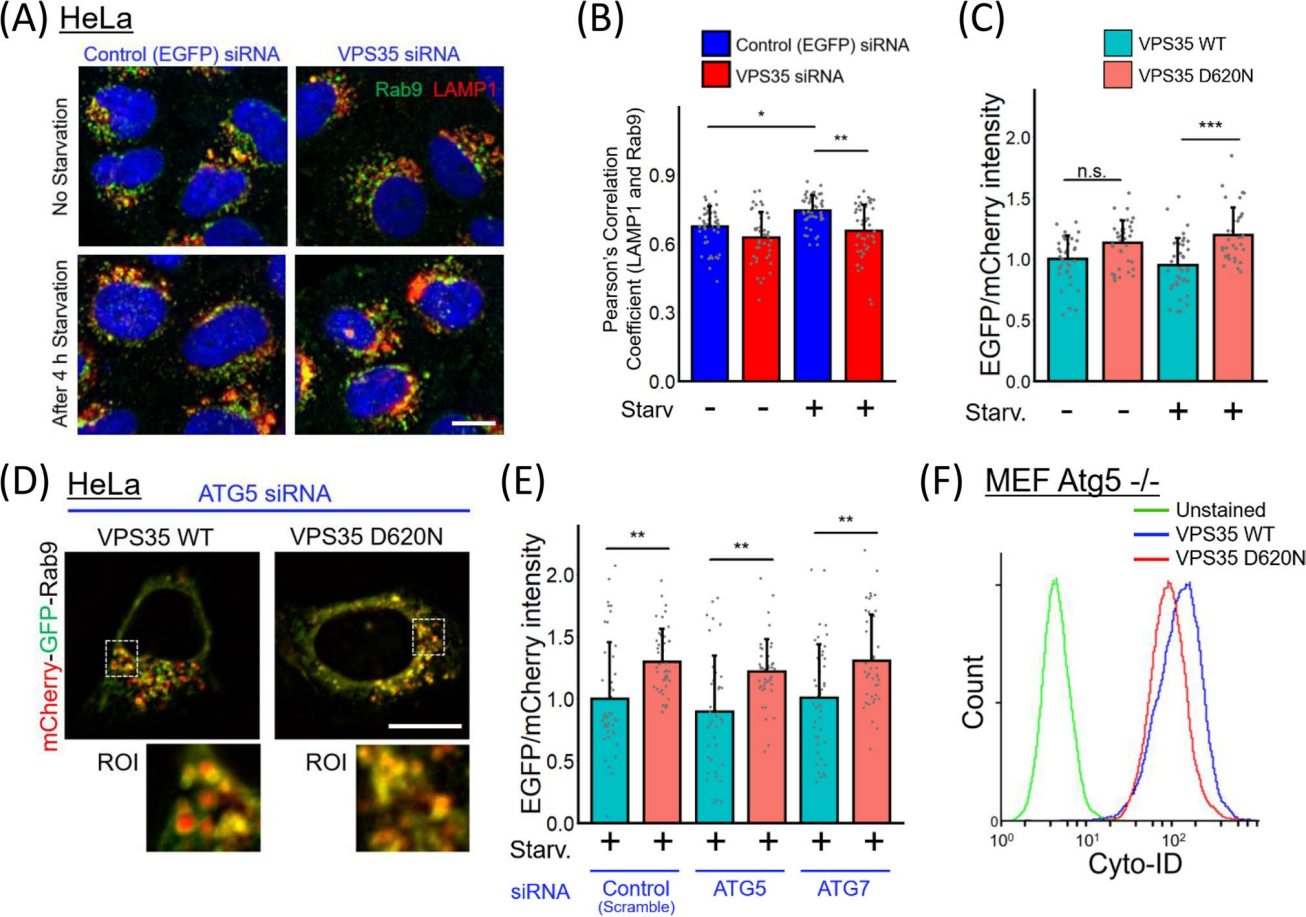
VPS35 D620N inhibits Rab9 localization to lysosomes. **A** Rab9 and LAMP1 localization in VPS35-knockdown cells compared to that in control cells. **B** Colocalization analysis of Rab9 and LAMP1. (*n* = 3 independent experiments; 43–49 cells in each group.) **C**, **E** The signal intensity ratio of EGFP/mCherry in Flag-VPS35 HeLa cells transiently expressing the mCherry-EGFP-Rab9 protein. Scrambled, ATG5, or ATG7 short interfering RNA (siRNA) was cotransfected with mCherry-EGFP-Rab9 in the cells shown in **E**. (*n* = 3 independent experiments; 35–45 cells in each group.) **D** Representative images showing Flag-VPS35 HeLa cells transiently expressing the mCherry-EGFP-Rab9 protein under ATG5 expression-knockdown conditions. Red puncta indicate mCherry-EGFP-Rab9 proteins in acidic organelles. **F** Atg5^−/−^ MEFs stably transfected with Flag-VPS35 WT or D620N were stained with Cyto-ID 4 h after starvation. Cyto-ID staining was analyzed using fluorescence-activated cell sorting. The bar graph represents the mean + SD. All data were analyzed using Kruskal–Wallis test, followed by multiple comparisons through the Bonferroni method. * p < 0.05, ** p < 0.01, and *** p < 0.001. Scale bars = 10 μm

While VPS35 is known to interact with Rab9 to regulate cellular trafficking [26], no study has evaluated the relationship between VPS35 and Rab9-dependent alternative autophagy. Therefore, we first cultured VPS35 siRNA-treated HeLa cells under starvation conditions to induce autophagy. After fixing the cells and labeling Rab9 and LAMP1, a lysosome marker, we found that the loss of VPS35 expression reduced the colocalization rate of Rab9 with LAMP1 (Figs. 1A, B and S1A). These data suggest that VPS35 is involved in Rab9 trafficking to lysosomes.

Using HeLa cells stably expressing WT Flag-VPS35 or the D620N mutant, we next investigated whether the VPS35 D620N mutation is involved in the regulation of alternative autophagy. After culture under starvation conditions, the D620N-expressing cells showed decreased colocalization between Rab9 and lysosomes (Fig. S2). Furthermore, we visualized Rab9 translocation to lysosomes using an mCherry-EGFP-Rab9 fusion protein, which loses EGFP fluorescence but not mCherry fluorescence under acidic conditions [13] (Fig. S3). WT Flag-VPS35 HeLa cells transiently transfected with mCherry-EGFP-Rab9 exhibited red puncta after starvation treatment; however, the red fluorescence intensity was decreased in D620N mutant cells (Fig. S4 and 1C). Moreover, this fluorescence pattern was preserved even when the expression of ATG5 or ATG7 (key molecules in conventional autophagy) were knocked down (Fig. 1D and E). In addition, the previous reports confirmed that alternative autophagy generates autophagosomes by fusing isolation membranes with vesicles from late endosomes as well as trans-Golgi [27, 28], therefore, we also examined whether syntaxin7 (a maker for late endosome) is associated with alternative autophagosomes. Using mCherry-EGFP-syntaxin7 fusion protein (Fig. S3), we found that the D620N mutation inhibited syntaxin7 to be translocated into lysosomes (Fig. S5).

To further confirm these findings, we next investigated autophagosome formation in Atg5^-/-^ MEFs [29] using Cyto-ID, which selectively labels autophagic vacuoles. A previous report demonstrated that Cyto-ID puncta are identical to autophagic vacuoles under ATG5-knockout conditions [30]. We confirmed that colocalization of autophagosome vacuoles and GS15 (a trans-Golgi marker) increased under ATG5-knockdown condition (Fig. S6). As we expected, autophagosome formation was impaired in Atg5^-/-^ MEFs stably transfected with VPS35 D620N (Fig. 1F). These results suggest that VPS35 D620N impairs alternative autophagy by inhibiting Rab9 localization to lysosomes.

### Estrogen treatment of VPS35 D620N neurons rescues alternative autophagy

**Fig. 2:**
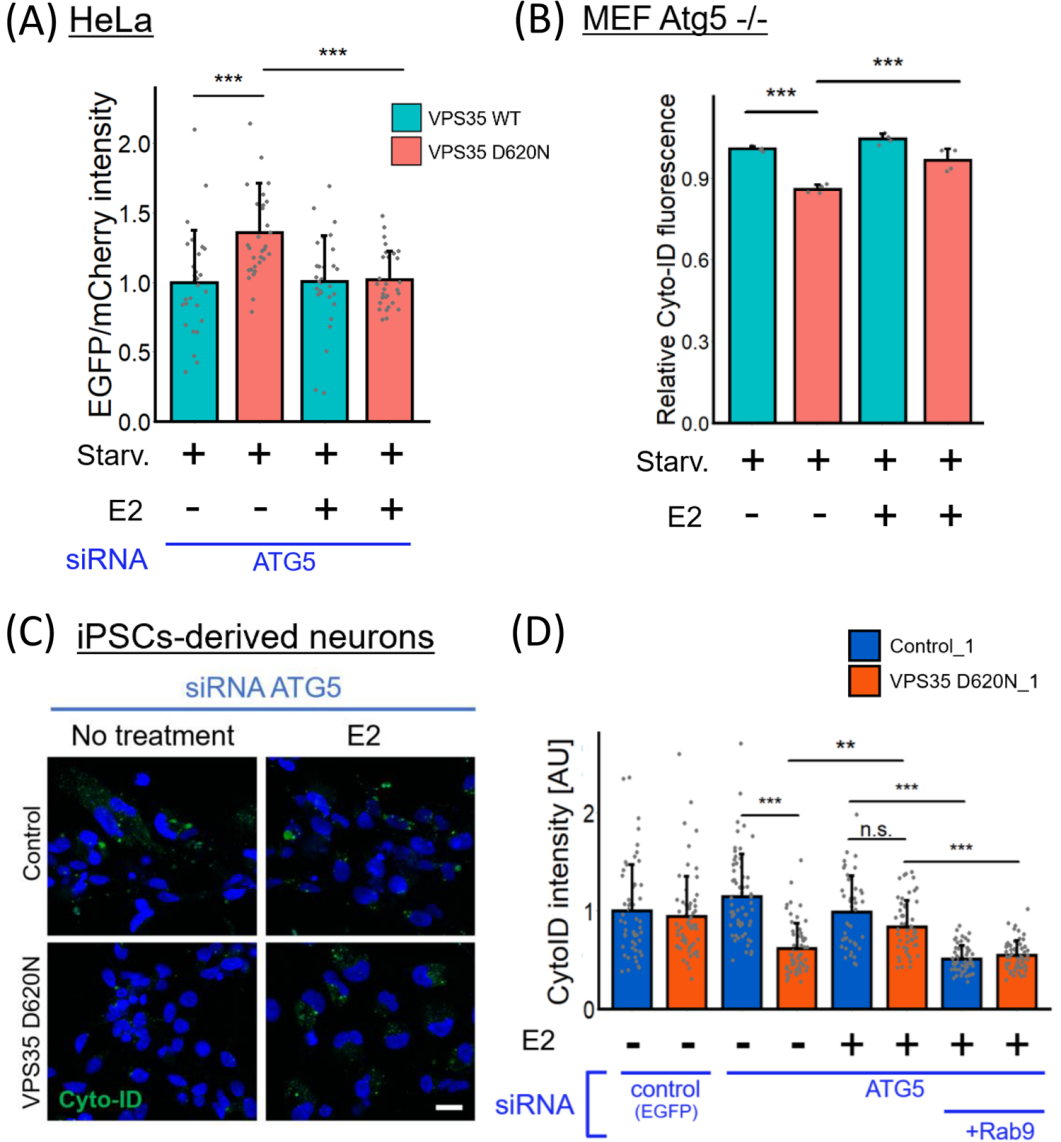
Estrogen activates alternative autophagy in VPS35 D620N cells. **A** Comparison of the signal intensity ratio of EGFP/mCherry in Flag-VPS35 HeLa cells transiently expressing the mCherry-EGFP-Rab9 protein under ATG5 expression-knockdown conditions with or without estrogen treatment. This experiment was simultaneously performed with the experiment described in Fig. 1E. (*n* = 3 independent experiments; 44–45 cells in each group.) **B** Atg5.^−/−^ MEFs stably transfected with Flag-VPS35 WT or D620N were treated with estrogen. After stained with Cyto-ID, mean fluorescence intensity was analyzed using fluorescence-activated cell sorting. (n = 4 independent experiments) **C** Analysis of alternative autophagy activity in iPSC-derived neurons through conventional autophagy suppression. Autophagic vacuoles in iPSC-derived neurons were analyzed by Cyto-ID. Scale bar = 10 μm. (D) The intensity of Cyto-ID fluorescence in each cell is shown. (*n* = 2 independent experiments; 43–62 cells in each group.) The bar graph represents the mean + SD. All data were analyzed using Kruskal–Wallis test, followed by multiple comparisons through the Bonferroni method. * p < 0.05, ** p < 0.01, and *** p < 0.001

The regulation of alternative autophagy is not fully understood; however, recent work has shown that 17*β*-estradiol (E2, estrogen) induces alternative autophagy [24], prompting us to examine the effect of estrogen on alternative autophagy in D620N mutant cells. Retromer dysfunction might have influence trafficking of estrogen receptors, such as estrogen receptor α, *β*, or G-protein-coupled estrogen receptor (GPER), however, there were no differences in estrogen receptor expression or subcellular localization between VPS35 WT and D620N cells (Fig. S7). As shown in Fig. 2A, we demonstrated that the relative red fluorescence intensity was increased after VPS35 D620N cell treatment with estrogen, suggesting that estrogen can reestablish alternative autophagy that had been disrupted by expression of the VPS35 D620N mutant. Similarly, autophagosome formation was upregulated by estrogen in Atg5 -/- MEFs stably transfected with VPS35 D620N (Fig. 2B).

We also sought to determine whether the D620N mutation impairs alternative autophagy in patient-derived iPSCs that were forced to differentiate into neurons. We generated iPSCs from the peripheral blood mononuclear cells of two PD patients with the D620N mutation in the VPS35 gene (VPS35 D620N_1 and 2) and a healthy control (Control_2) as previously described [16] and used 201B7 cells, namely, Control_1 cells. After 12–14 days of differentiation, there were no differences in the ratio of MAP2-positive cells and tyrosine hydroxylase (TH, a dopaminergic neuronal marker)-positive cells between the control and VPS35 D620N groups (Fig. S8).

To elicit alternative autophagy in iPSC-derived neurons, we suppressed conventional autophagy by ATG5 siRNA (Fig. S1B). As shown in Fig. 2C and D, the Cyto-ID fluorescence intensity was significantly decreased in ATG5-knockdown iPSC-derived neurons carrying the VPS35 D620N mutation compared with ATG5-knockdown VPS35 WT neurons, suggesting that compensation for alternative autophagy was insufficient in the VPS35 D620N iPSC-derived neurons. Moreover, estrogen increased the number of autophagic vacuoles in ATG5 knockdown VPS35 D620N mutant neurons; however, Rab9 expression knockdown eliminated the effect of estrogen on alternative autophagy induction (Fig. 2C, D). Taken together, our results indicate that estrogen can rescue the alternative autophagy that had been impaired in VPS35 D620N mutant cells.

### The VPS35 D620N mutation alters the dynamics of Rab9 vesicles

**Fig. 3:**
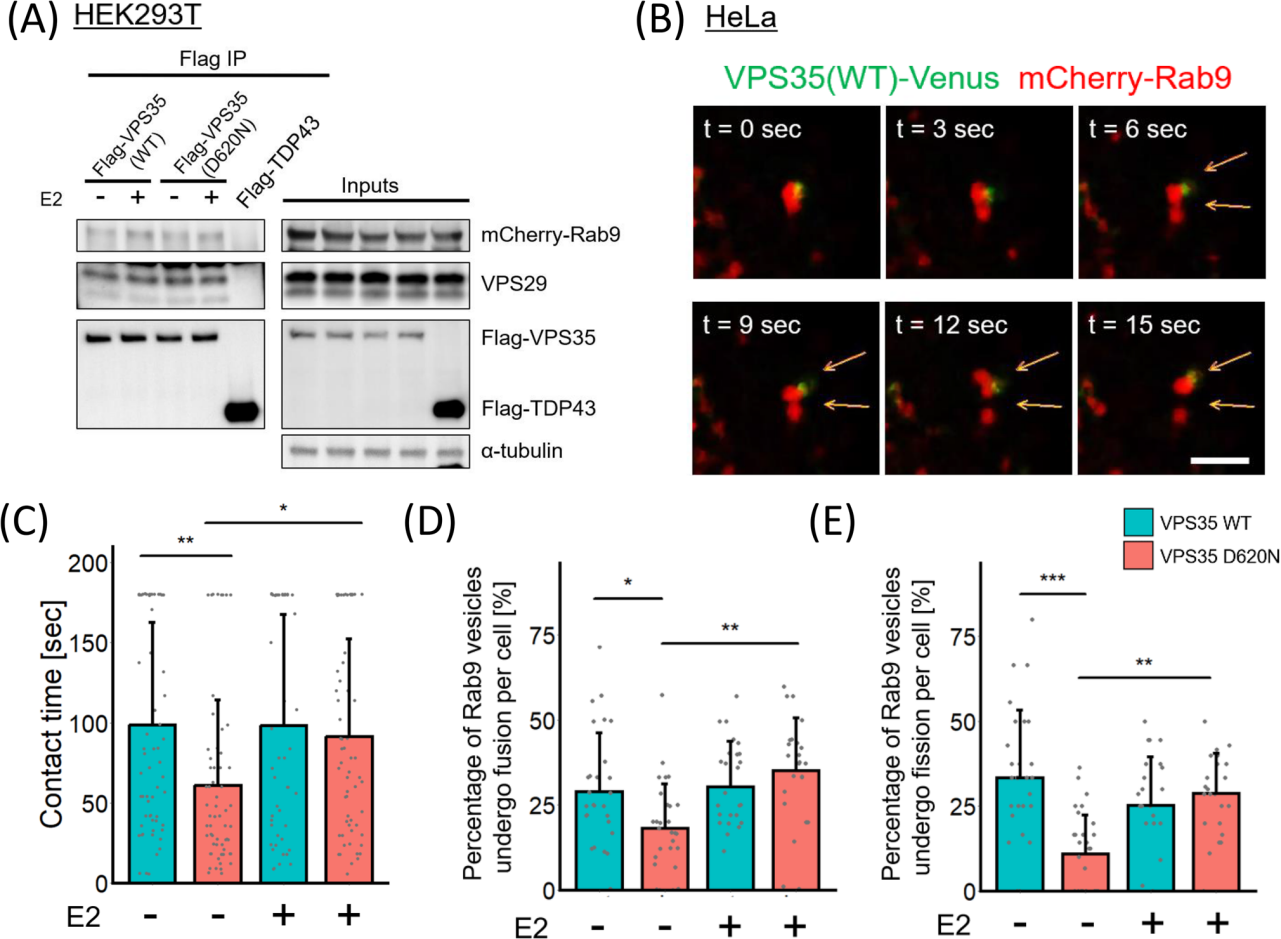
VPS35 D620N alters the dynamics of Rab9 vesicles. **A** mCherry-Rab9 co-immunoprecipitated with Flag-VPS35 in HEK293T cells. Flag-TDP43 was used as control. **B** Live-cell imaging showing VPS35 (WT)-Venus and mCherry-Rab9 in HeLa cells. VPS35 colocalizes with budding mCherry-Rab9 vesicles in HeLa cells. Scale bar = 1 μm. **C** Relative frequency distribution of the duration of VPS35 and Rab9 contact. (n = 3 independent experiments; 48–66 contacts in each group.) **D**, **E** The mean of the percentage of Rab9 vesicles undergoing fusion or fission in the 3-min videos. (n = 3 independent experiments; 26–30 cells in each group.) All data were analyzed using Kruskal–Wallis test, followed by multiple comparisons through the Bonferroni method. * p < 0.05, ** p < 0.01, and *** p < 0.001. The bar graph represents the mean + SD

The result that VPS35 D620N impaired alternative autophagy prompted us to examine how the D620N mutation influences Rab9 trafficking. Flag-trap isolation of Flag-VPS35 revealed that the D620N mutation or estrogen had no effect on VPS35 binding to Rab9 (Fig. 3A). To further elucidate the mechanisms by which the D620N mutation impairs Rab9-dependent alternative autophagy and estrogen promotes the induction of alternative autophagy in VPS35 D620N cells, we coexpressed VPS35-Venus and mCherry-Rab9 in HeLa cells and performed time-lapse observation of both VPS35 and Rab9. As previously reported, VPS35 expression coincided with budding Rab9 vesicles [26] (Fig. 3B). In contrast to that of WT VPS35-Venus, the expression of D620N VPS35-Venus led to a significantly decreased VPS35-Rab9 contact duration (Fig. 3C, S9, Video. S1, and S2), suggesting that VPS35 D620N altered the contact dynamics between VPS35- and Rab9-carrying vesicles. In addition, estrogen treatment of VPS35 D620N cells increased the duration of VPS35-Rab9 contact (Fig. 3C).

We also counted Rab9 vesicles that underwent fission and fusion for 3 min. The fusion and fission frequency of Rab9 vesicles was significantly lower in the VPS35 D620N cells than in the WT cells (Video. S3 and S4) and was significantly increased after estrogen treatment (Fig. 3D and E). Furthermore, in the context of retromer-mediated cargo sorting, tubular endosomes have been characterized as recycling endosomal compartments [31]. Hence, we investigated the formation of Rab9-positive tubular endosomes by measuring the total tubule formation lengths with Rab9 vesicles in HeLa stable cell lines (Fig. S10A). We confirmed that tubule formation with EGFP-Rab9 vesicles was accompanied by expression of mCherry-Rab10, a novel protein predominantly localized at tubular endosomes (Fig. S10B, Video S5–S10) [32]. These results showed that the total tubule length in VPS35 D620N cells was significantly shorter than that in the WT cells (Fig. S10C, D). Intriguingly, estrogen significantly decreased the total tubule length in the WT cells (Fig. S10D).

### Estrogen does not restore the impairment of VPS35-WASH complex interaction in D620N mutant cells

**Fig. 4:**
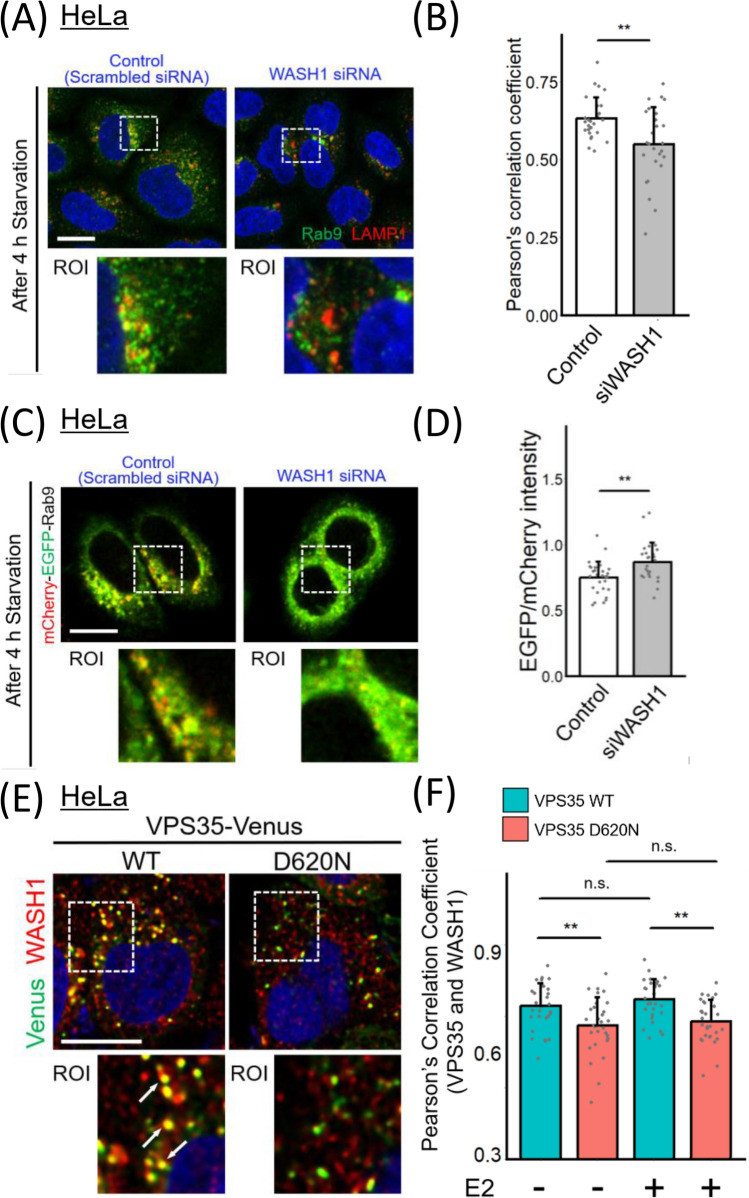
WASH1 is involved in Rab9 subcellular localization, but estrogen has no effect on WASH1-VPS35 colocalization. **A** Rab9 and LAMP1 localization in WASH1-knockdown cells compared to that in control cells. **B** Colocalization analysis of Rab9 and LAMP1. (*n* = 3 independent experiments; 30 cells in each group.) The data were analyzed using Student’s *t* test. **C** HeLa cells transiently expressing the mCherry-EGFP-Rab9 protein under WASH1 knockdown conditions compared to control cells. **D** The signal intensity ratio of EGFP/mCherry of HeLa cells in **C**. (n = 3 independent experiments; 30 cells in each group.) The data were analyzed using Student’s *t* test. **E** VPS35 and WASH1 localization in HeLa cells transiently transfected with VPS35 (WT or D620N)-Venus. Arrows represent colocalization of VPS35 and WASH1. **F** Colocalization analysis of VPS35 and WASH1. (n = 3 independent experiments; 30 cells in each group.) The data were analyzed using Kruskal–Wallis test, followed by multiple comparisons through the Bonferroni method. * p < 0.05, ** p < 0.01, and *** p < 0.001. The bar graph represents the mean + SD. Scale bars = 10 μm

The major defect of the D620N mutation is thought to lie in its association with the Wiskott-Aldrich syndrome and SCAR homolog (WASH) complex, which plays a critical role in endosomal vesicle budding [9, 33]. As it is reported that the WASH complex localizes to the budding site of Rab9 endosomes [26], we first examined how knockdown of WASH1 (Fig. S1C), one of the components of WASH complex, affect Rab9 trafficking. As shown in Fig. 4A and B, cells treated with WASH1 siRNA showed decreased colocalization between Rab9 and LAMP1. Furthermore, HeLa cells transfected with mCherry-EGFP-Rab9 exhibited reduced red puncta under WASH1 suppression condition (Fig. 4C and D). These results suggest that WASH complex is involved in Rab9 localization to lysosomes.

We next investigated whether estrogen affect the interaction between VPS35 and WASH complex. As we expected [9, 33], the colocalization of VPS35 and WASH1 was impaired in the D620N mutated HeLa cells, however, estrogen did not restore the colocalization of VPS35 and WASH1 (Fig. 4E and F). Therefore, estrogen may restore alternative autophagy defects in the D620N cells by acting on mechanisms other than the VPS35-WASH complex interaction.

### Phosphorylation of Rab9 at S179 is involved in the regulation of alternative autophagy by estrogen

**Fig. 5:**
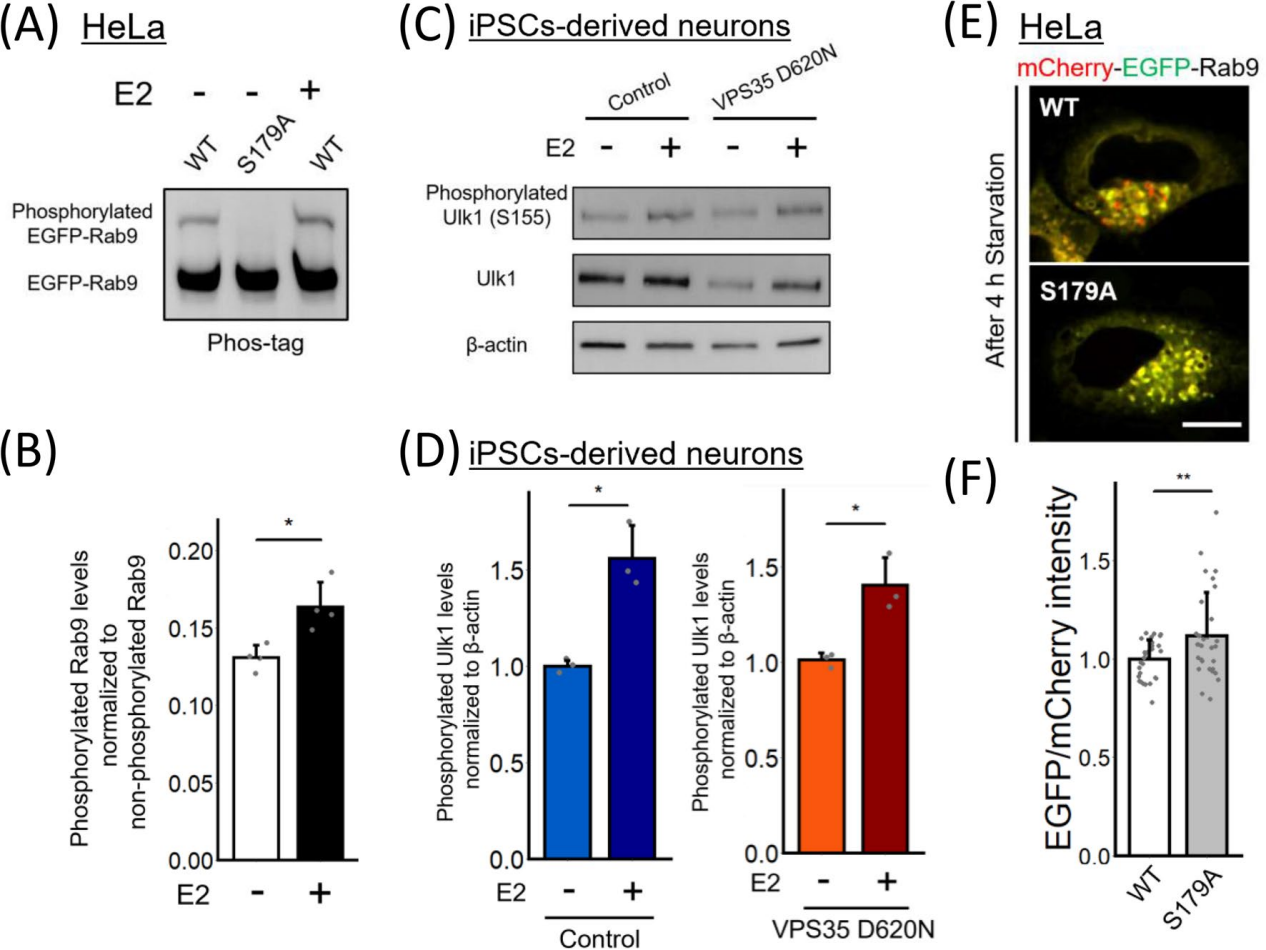
Rab9 phosphorylation at S179 is promoted by estrogen and is significant in the regulation of alternative autophagy. **A** HeLa cells transiently expressing EGFP-Rab9 WT or S179A were treated with estrogen. The lysate was analyzed by SDS–PAGE with Phos-tag. **B** Comparison of phosphorylated Rab9 levels with or without estrogen treatment in HeLa cells transiently expressing EGFP-Rab9. (n = 4 independent experiments.) **C**, **D** Comparison of phosphorylated Ulk1 levels in iPSC-derived neurons with or without estrogen treatment. (n = 3 independent experiments.) **E** Representative images showing HeLa cells transiently expressing the mCherry-EGFP-Rab9 WT or S179A protein. The number of red puncta was decreased in cells expressing mCherry-EGFP-Rab9 S179A. Scale bar = 10 μm. **F** Comparison of the signal intensity ratio of EGFP/mCherry in HeLa cells expressing mCherry-EGFP-Rab9 WT or S179A. (n = 2 independent experiments; 31 cells in each group.) All the data were analyzed using Student’s *t* test. * p < 0.05, ** p < 0.01, and *** p < 0.001. The bar graph represents the mean + SD

Next, we investigated the molecular mechanism through which estrogen altered Rab9 vesicle trafficking. Since a previous study has shown that SIRT1/LKB1/AMPK/Ulk1 pathway activation was accelerated by estrogen and that estrogen-mediated effects on alternative autophagy were abolished by Ulk1 expression knockdown [24], we hypothesized that these pathway molecules are involved in the regulation of Rab9 trafficking dynamics. Another study has shown that AMPK phosphorylates Ulk1 at S555 and that phosphorylated Ulk1 (S555) then phosphorylates Rab9 at S179 to promote alternative autophagy [34]. Therefore, we first investigated whether estrogen promotes Rab9 phosphorylation at S179. A previous study has demonstrated that phosphorylated Rab9 can be identified as a unique band in Phos-tag SDS PAGE [34]. In our present study, we found that the phosphorylation of Rab9 at S179 was significantly increased in estrogen-treated HeLa cells transiently transfected with EGFP-Rab9 (Fig. 5A and B). Similar levels of Rab9 phosphorylation were found in both VPS35 WT and D620N cells (Fig. S11). Furthermore, estrogen increased not only the level of Ulk1 [35] but also that of phosphorylated Ulk1 (S555) in both VPS35 WT and D620N iPSC-derived neurons (Fig. 5C and D). These data suggest that estrogen promotes the phosphorylation of Rab9 at S179 mediated through Ulk1 phosphorylation.

We next examined whether a point mutation at S179 affects the induction of alternative autophagy by transiently transfecting HeLa cells with mCherry-EGFP-Rab9 WT or S179A. After starvation treatment, the relative red fluorescence intensity was decreased in the HeLa cells expressing mCherry-EGFP-Rab9 S179A (Fig. 5E and F). Taken together, these results suggest that Rab9 phosphorylation at S179 is involved in alternative autophagy and is promoted by estrogen treatment. We also examined whether the phosphorylation of Rab9 at S179 affect Rab9-WASH complex interaction, however, the S179A mutation did not have influence on Rab9-WASH1 colocalization (Fig.S12).

### The neuroprotective effect of estrogen depends on Rab9 in patient-derived VPS35 D620N neurons

**Fig. 6:**
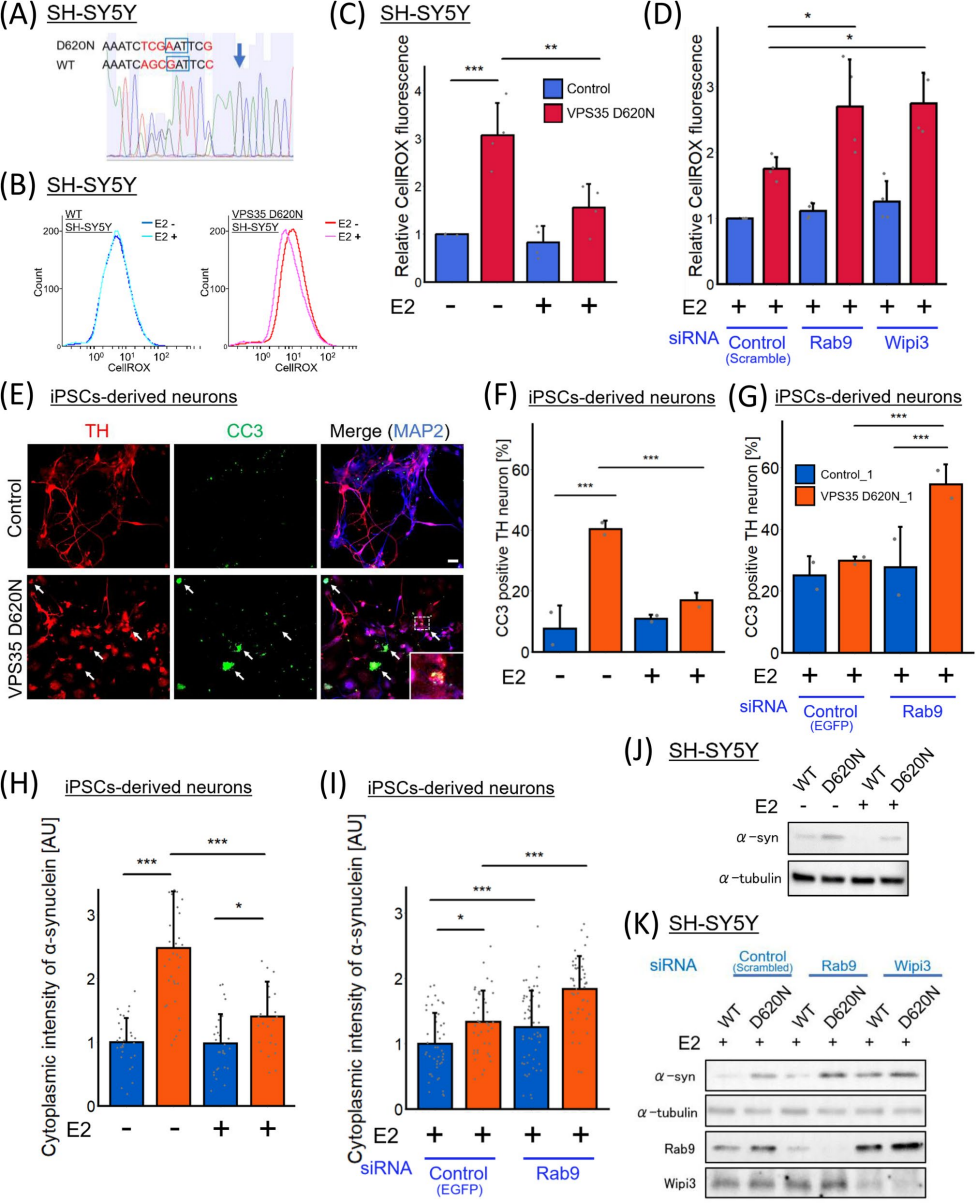
The neuroprotective effect of estrogen depends on Rab9 in patient-derived VPS35 D620N neurons. **A** Using CRISPR-Cas9 engineering, we generated two clones of SH-SY5Y cells carrying the VPS35 D620N mutation. Through Sanger sequencing, the presence of the D620N mutation, involving a substitution from GAT to AAT (indicated by the encompassing blue lines), in one of the two VPS35 alleles was confirmed. Blue arrow represents a Cas9-cleavage site. The sequencing outcome for D620N #2 is depicted in Fig.S14C. **B** The differentiated SH-SY5Y with WT or D620N VPS35 were stained with CellROX and analyzed using fluorescence-activated cell sorting. **C**, **D** The resulting fluorescence intensity of CellROX in each cell is presented. Scrambled, Rab9, or Wipi3 siRNA was transfected in the cells shown in **D**. The dataset includes a total of four experiments; D620N #1 is represented by n = 2, and D620N #2 by n = 2. **E** Results of an apoptosis analysis based on cleaved caspase-3 (CC3) expression in differentiated dopaminergic neurons. Scale bar = 20 μm. **F**, **G** Frequencies of CC3-positive neurons in each group. The data were analyzed by chi-squared test. (n = 2 independent experiments; 82–137 cells in each group.) **H**, **I** Immunostaining for α-synuclein of differentiated dopaminergic neurons derived from control and VPS35 D620N iPSCs and the resulting quantitation of the cytoplasmic intensity of α-synuclein were performed (n = 2 independent experiments; 74–90 cells in each group.). Scrambled or Rab9 siRNA were transfected in the cells shown in **I**. **J**, **K** Comparison of α-synuclein levels with or without estrogen treatment in the differentiated WT or D620N #1 SH-SY5Y cells. Scrambled, Rab9, or Wipi3 siRNA was transfected in the cells shown in (K). The data were analyzed using Kruskal–Wallis test, followed by multiple comparisons through the Bonferroni method. * p < 0.05, ** p < 0.01, and *** p < 0.001. The bar graph represents the means + SD

Recent experimental and clinical studies have suggested that estrogen may exert a protective effect on dopaminergic neurons in PD [36, 37]. Therefore, considering our previous discovery revealing that the patient-derived VPS35 D620N iPSC model produced vulnerable dopaminergic neurons with accumulated *α*-syn [16], we quantified the effect of estrogen on damaged patient-derived VPS35 D620N neurons. We quantified ROS production in iPSC-derived VPS35 D620N neurons from a patient and control iPSC-derived neurons from a healthy individual using CellROX. Whereas the VPS35 D620N iPSC-derived dopaminergic neuron population exhibited a higher percentage of ROS-positive cells than the population derived from the healthy individual, estrogen reduced the ROS accumulation in the VPS35 D620N iPSC-derived dopaminergic neurons (Fig. S13). Furthermore, we attempted to validate this outcome within CRISPR/Cas9-engineered VPS35 D620N mutant cells. Following the protocols outlined by Ma et al. [19], we generated two clones of SH-SY5Y cells carrying the heterozygous VPS35 D620N mutation (Fig. 6A and S14). Consequently, differentiated VPS35 D620N mutant SH-SY5Y cells also exhibited higher ROS accumulation than WT cells (Fig. 6B, C), and estrogen mitigated the oxidative stress in the VPS35 D620N mutant (Fig. 6C). To address whether the antioxidative effect of estrogen depends on alternative autophagy, we also performed the experiments under alternative autophagy knockdown condition. The reduction in ROS levels induced by estrogen was diminished under Rab9-knockdown conditions, not only in patient-derived VPS35 D620N neurons (Fig. S13C), but also in VPS35 D620N SH-SY5Y cells (Fig. 6D). This result was validated by knocking down Wipi3, a molecule essential for alternative autophagy (Fig. 6D) [13].

Moreover, we found a significant increase in the cleaved caspase 3 (CC3)-positive cell subpopulation of the patient-derived VPS35 D620N dopaminergic neuron population, and this effect was abrogated by estrogen treatment (Fig. 6E, F, and S15A). These data showed that estrogen treatment attenuated the damage to PD neurons carrying the VPS35 D620N mutation. In addition, estrogen reduced α-syn accumulation in iPSC-derived VPS35 D620N dopaminergic neurons (Fig. 6H and S15C). Rab9 expression knockdown increased the apoptosis rate of patient-derived VPS35 D620N neurons even after estrogen treatment (Fig. 6G and S15B). In an α-syn accumulation evaluation, Rab9 expression knockdown promoted α-syn accumulation in both control and VPS35 D620N-derived dopaminergic neurons (Fig. 6I and S15D). These results were also confirmed in the CRIPSR/Cas9-generated VPS35 D620N mutant SH-SY5Y cells using western blotting (Fig. 6J, K and S16), and suggest that the neuroprotective effect of estrogen depends on Rab9 expression in patient-derived VPS35 D620N neurons and that alternative autophagy plays an important role in α-syn clearance from dopaminergic neurons.

## Discussion

The mechanism through which retromer dysfunction affects neurodegeneration in PD is not fully understood. In the present study, we generated a dopaminergic neuron-enriched culture by inducing patient-derived VPS35 D620N expression in iPSCs. We demonstrated that this mutation impairs Rab9-dependent alternative autophagy. Furthermore, we found that estrogen triggers the activation of alternative autophagy and provides neuroprotection to dopaminergic neurons derived from patients with the VPS35 D620N mutation, a process that is dependent on Rab9 expression.

The retromer complex is crucial for proper trafficking of autophagy-related proteins, such as LAMP2a, which is involved in chaperone-mediated autophagy [38], and ATG9, which is necessary for conventional autophagy induction [9]. The D620N mutation in VPS35 leads to the failure of cathepsin D delivery to the lysosome, resulting from the abnormal retromer-dependent endosomal sorting of CI-MPR [8]. In fact, impaired autophagic flux and decreased lysosomal mass and size were confirmed in patient-derived dopaminergic VPS35 D620N neurons [11]. In the present study, we are the first to reveal that the D620N mutation in VPS35 caused dysregulated intracellular trafficking of Rab9 and impaired alternative autophagy induction. Taking into consideration a previous report showing that the retromer complex is involved in autophagosome formation in conventional autophagy, VPS35 may be crucial for autophagosome formation in both conventional and alternative autophagy.

Our findings indicating that alternative autophagy activation can confer neuroprotection to VPS35 D620N neurons may have implications for the association between PD pathogenesis and alternative autophagy. Whereas no universal marker of alternative autophagy exists, one of the most reliable techniques for confirming alternative autophagy is the evaluation of autophagosome in ATG5- or ATG7-depleted cells, in which residual canonical autophagy does not occur [39]. The role played by alternative autophagy in neurodegenerative diseases has not yet been elucidated; however, recent studies have revealed a close relationship between alternative autophagy and the maintenance of neuronal cells. For example, knocking out the expression of Wipi3, a molecule essential for alternative autophagy, caused neurodegeneration similar to that in static encephalopathy of childhood with neurodegeneration in adulthood (SENDA) [13]. Another report suggested that stalling canonical autophagy induction promoted neuronal homeostatic maintenance mediated by alternative autophagy in mice with dentatorubral-pallidoluysian atrophy (DRPLA) [14]. Furthermore, baseline levels of Rab9 protein were found to be significantly lower in neurons derived from patients with PD caused by the Miro1 R272Q mutation, suggesting that pathological changes in alternative autophagy may be evident in both Miro1 R272Q mutant and VPS35 D620N mutant models of monogenic PD [40]. Further experiments with several PD models are needed to elucidate the relationship between PD pathophysiology and alternative autophagy.

To date, several studies have reported the neuroprotective effect of estrogen on dopaminergic neurons in PD. The onset of PD is delayed in women with prolonged endogenous estrogen exposure [41], and exogenous estrogen may improve PD symptom severity [42]. Experimental research with manganese- or 6-hydroxydopamine-induced dopaminergic loss in animal PD models [43, 44] has supported the idea that estrogen confers a neuroprotective effect. Despite the epidemiological and experimental evidence showing the neuroprotective effect of estrogen, the molecular mechanism is not well understood. Moreover, the role of autophagy in estrogen-mediated neuroprotection has gained attention, particularly in a recent PD rat model study. This study demonstrated that estrogen induces expression of Ulk1 gene, which regulates not only conventional but also alternative autophagy [35]. The present study suggests that estrogen can rescue dopaminergic neurons by inducing alternative autophagy in PD.

Many studies have addressed α-syn degradation mechanisms, including the ubiquitin–proteasome system and autophagy [45], and autophagy is currently considered to be the main route of α-syn clearance [46]. Hanss et al. [11] revealed that rapamycin, an activator of conventional autophagy, reduces α-syn accumulation in VPS35 D620N dopaminergic neurons. However, a recent study has shown that inhibition of conventional autophagy by silencing ATG5 expression did not accelerate α-syn-induced toxicity [47], suggesting that an alternative degeneration mechanism may be triggered by accumulated α-syn. As the present study demonstrated that inhibition of alternative autophagy caused α-syn accumulation and dopaminergic neuronal death, alternative autophagy may be another route for α-syn degradation. While conventional autophagy certainly contributes to α-syn turnover, our findings suggest a significant role for alternative autophagy in α-syn clearance. Nonetheless, our study has limitations, notably the lack of in vivo evidence and an analysis of α-syn phosphorylation in iPSC-derived neurons. These aspects underscore the need for further research to comprehensively understand the role of alternative autophagy in α-syn degradation mechanisms.

We have shown that fusion and fission of Rab9 vesicles were inhibited in VPS35 D620N cells. Dong et al. demonstrated that retromer is involved in the budding of Rab9-positive endosomes via a WASH complex machinery [26]. One of the molecular effects of the D620N mutation is the reduction of retromer association with the WASH complex [9], indicating that reduced association of mutant VPS35 with WASH complex might result in the decrease in fusion/fission frequency of Rab9 vesicles. Endosome tubule formation is considered a key process in cargo protein recycling [48]. The retromer trimer functions with SNX-BAR proteins to package cargo proteins into tubular structures for transport to the TGN or plasma membrane [49]. Notably, Rab9 is known to play an important role in the retrograde transport of CI-MPR [50]. Based on this fact, it is possible that estrogen may improve lysosome function by improving the retrograde transport of CI-MPR as estrogen improved the VPS35-Rab9 interaction in the D620N cells. However, we found that the decrease in tubule formation in VPS35 D620N cells was not accelerated by estrogen. As this observation could be due to estrogen-induced fission of Rab9 endosomes, further studies are needed to confirm the effect of estrogen on retrograde transport by the retromer complex.

Emerging studies have suggested that the phosphorylation of Rab proteins may control Rab membrane-trafficking functions [51]. We found that phosphorylation of Rab9 at S179 was significantly increased by estrogen treatment and that this phosphorylation was involved in the regulation of alternative autophagy; however, the mechanism through which Rab9 phosphorylation is related to the modulated Rab9 trafficking remains unclear. In particular, the observation that estrogen enhances the VPS35-Rab9 interaction in the D620N mutant cells, while not affecting the VPS35-WASH complex interaction, points to a possible role of Rab9 phosphorylation in modulating retromer-endosome binding, which should be clarified in future studies. It is possible that phosphorylation of the C-terminal domain in Rab proteins may affect Rab interaction with GDP/GTP dissociation inhibitor (GDI) and thus regulate the extraction and reinsertion cycle of Rabs [51].

## Conclusions

In summary, our data suggest that alternative autophagy might be important in the pathophysiology of PD with the VPS35 D620N mutation and that estrogen can rescue alternative autophagy dysfunction. Exploring the mechanisms involved in alternative autophagy may allow the development of novel therapeutic strategies in PD.

### Supplementary Information

Below is the link to the electronic supplementary material.

Supplementary file1 (DOCX 16718 KB)

Supplementary file2 (DOCX 14 KB)

Supplementary file3 (PPTX 16012 KB)

Supplementary file4 (PPTX 6079 KB)

## Data Availability

The datasets analyzed during the current study are available from the corresponding author on reasonable request.

## Abbreviations

CC3: Cleaved caspase-3
CI-MPR: Cation-independent mannose-6-phosphate receptor
E2: Estrogen, 17β-estradiol
iPSC: Induced pluripotent stem cell
PD: Parkinson’s disease
ROS: Reactive oxygen species
TGN: Trans-Golgi network
VPS35: Vacuolar protein sorting
