# Supplementary material for: The impact of VPS35 D620N mutation on alternative autophagy and its reversal by estrogen in Parkinson's disease
Source: Cell Mol Life Sci. 2024 Feb 27;81(1):103. doi: 10.1007/s00018-024-05123-4 (PMC10896810; doi:10.1007/s00018-024-05123-4)
Supplement: Supplementary file 1 — Supplementary file1 (DOCX 16718 KB) [file 18_2024_5123_MOESM1_ESM.docx]

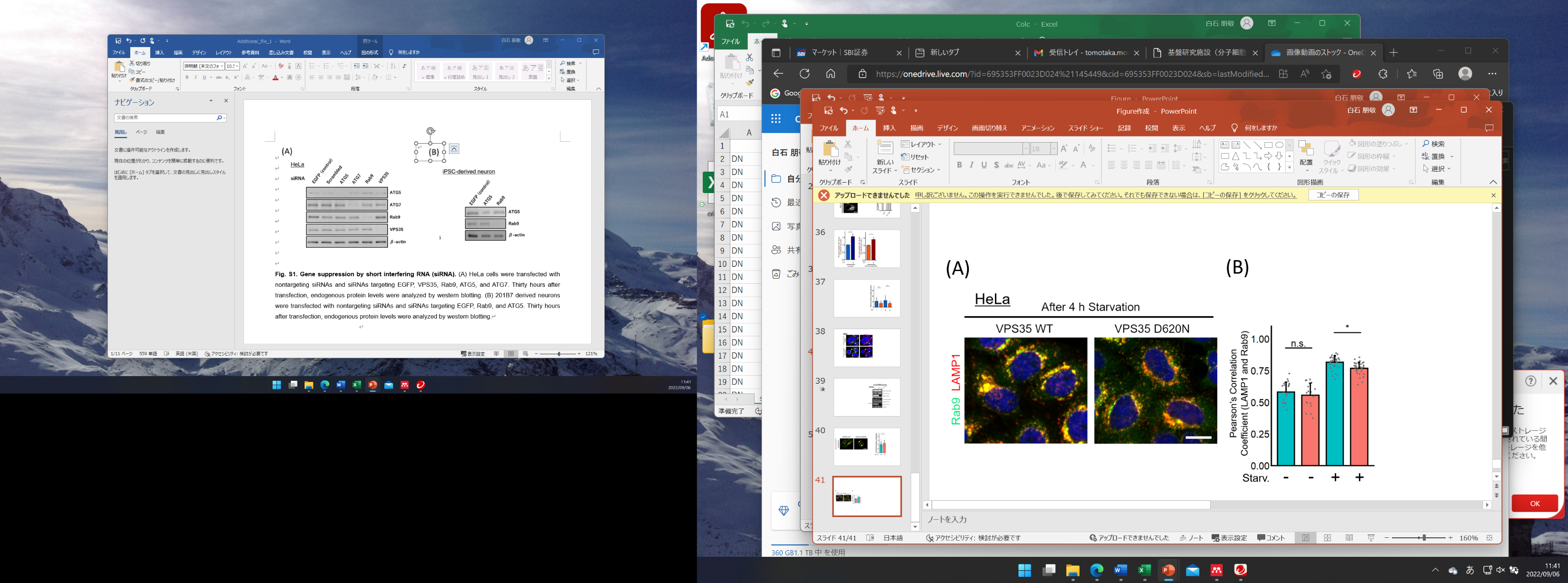

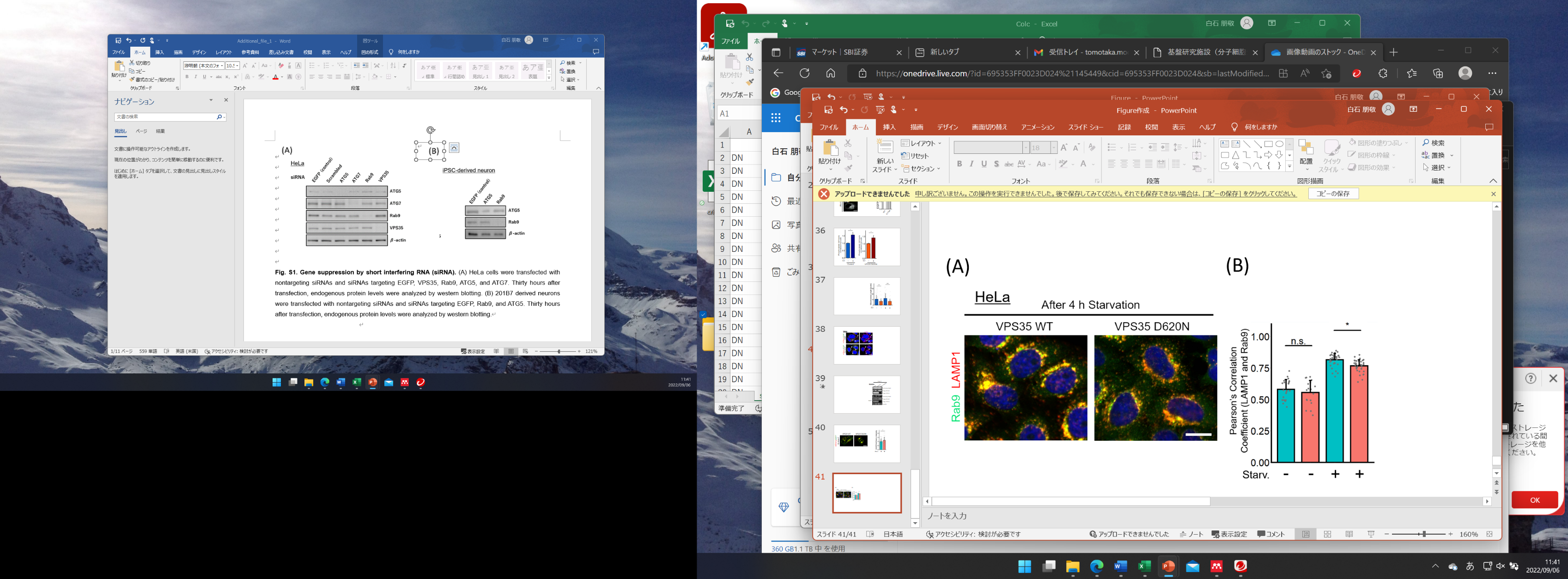

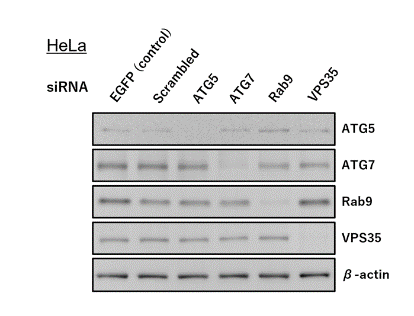


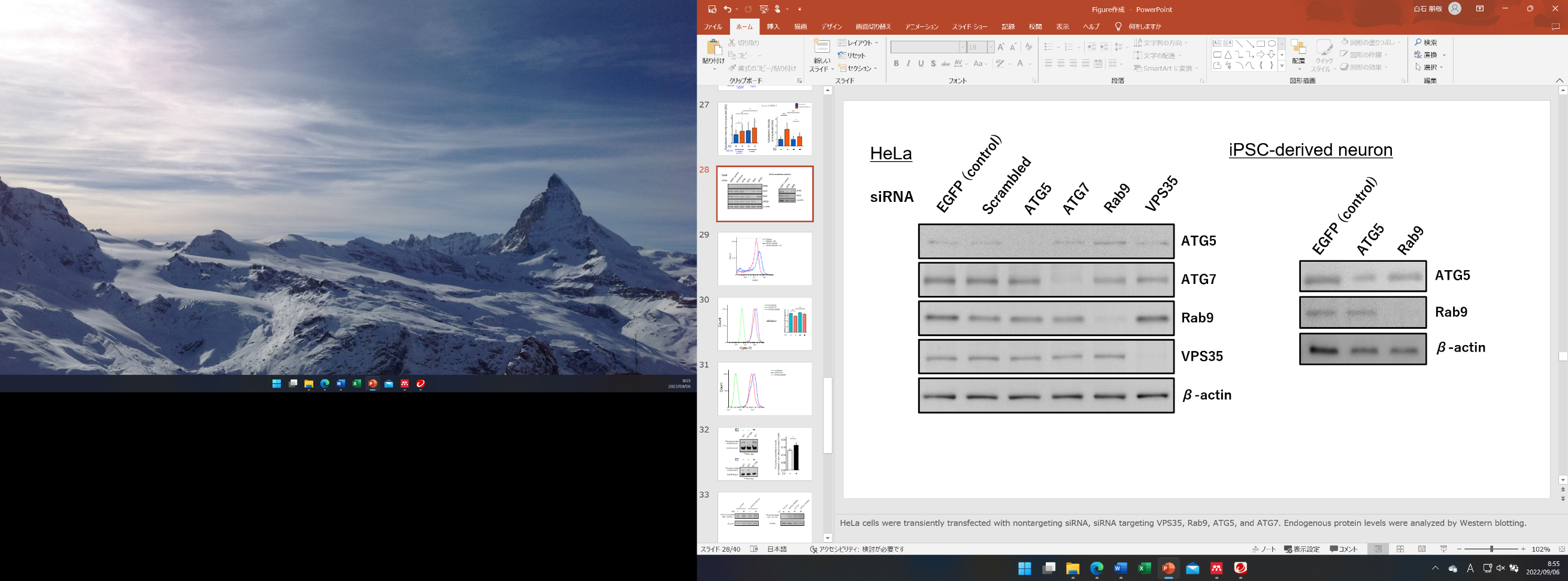


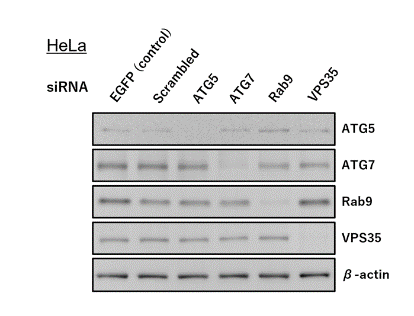


**
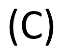
**

**
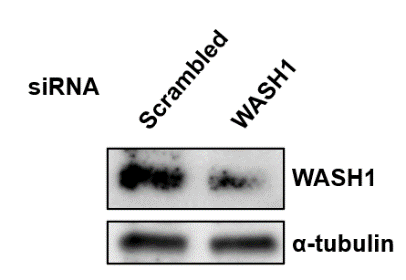
**

**Fig. S1 Gene suppression by short interfering RNA (siRNA).** (A) HeLa cells were transfected with nontargeting siRNAs and siRNAs targeting EGFP, VPS35, Rab9, ATG5, and ATG7. Thirty hours after transfection, endogenous protein levels were analyzed by western blotting. (B) 201B7 derived neurons were transfected with nontargeting siRNAs and siRNAs targeting EGFP, Rab9, and ATG5. Thirty hours after transfection, endogenous protein levels were analyzed by western blotting. (C) HeLa cells were transfected with nontargeting siRNAs and siRNAs targeting WASH1


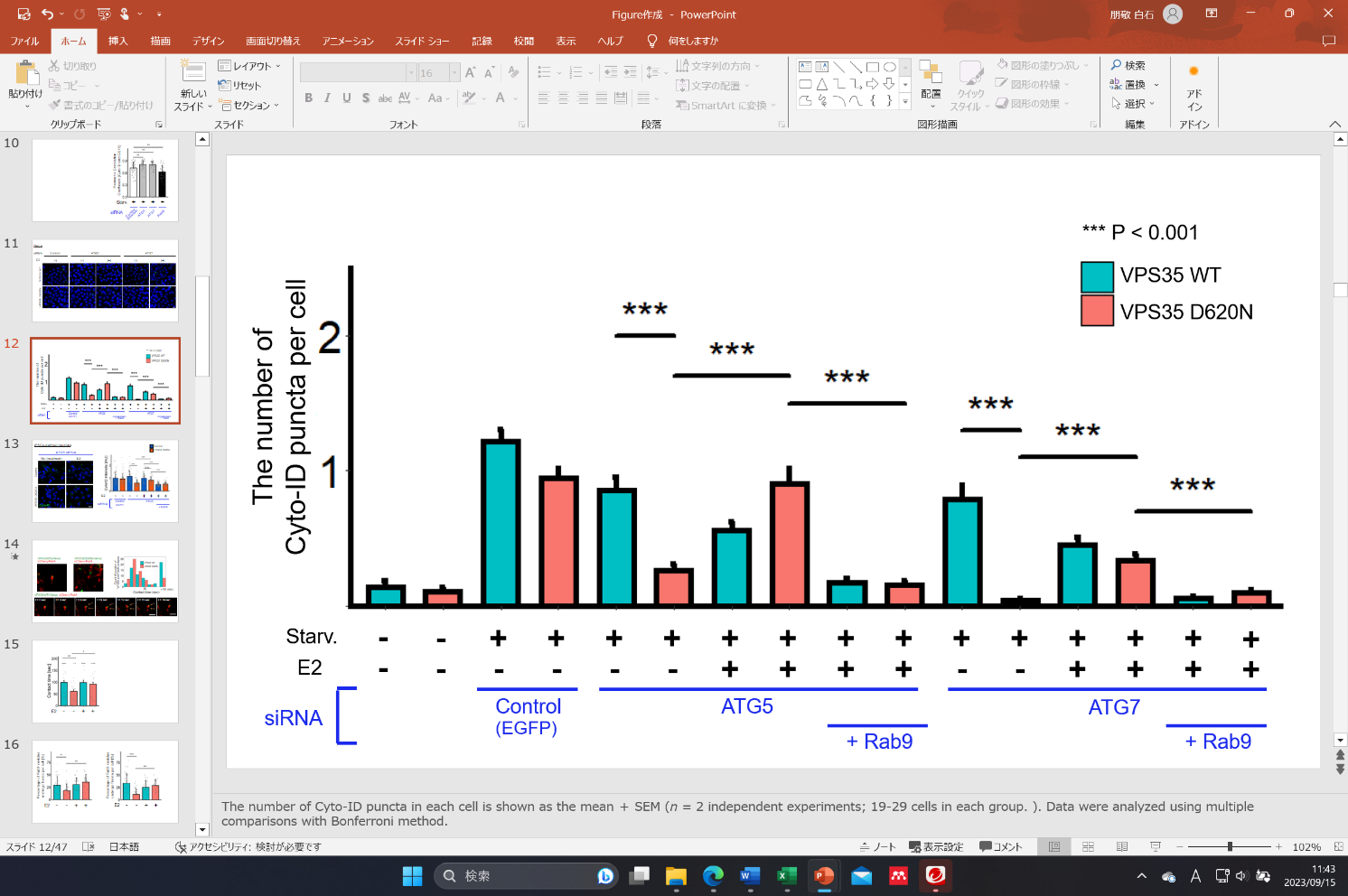

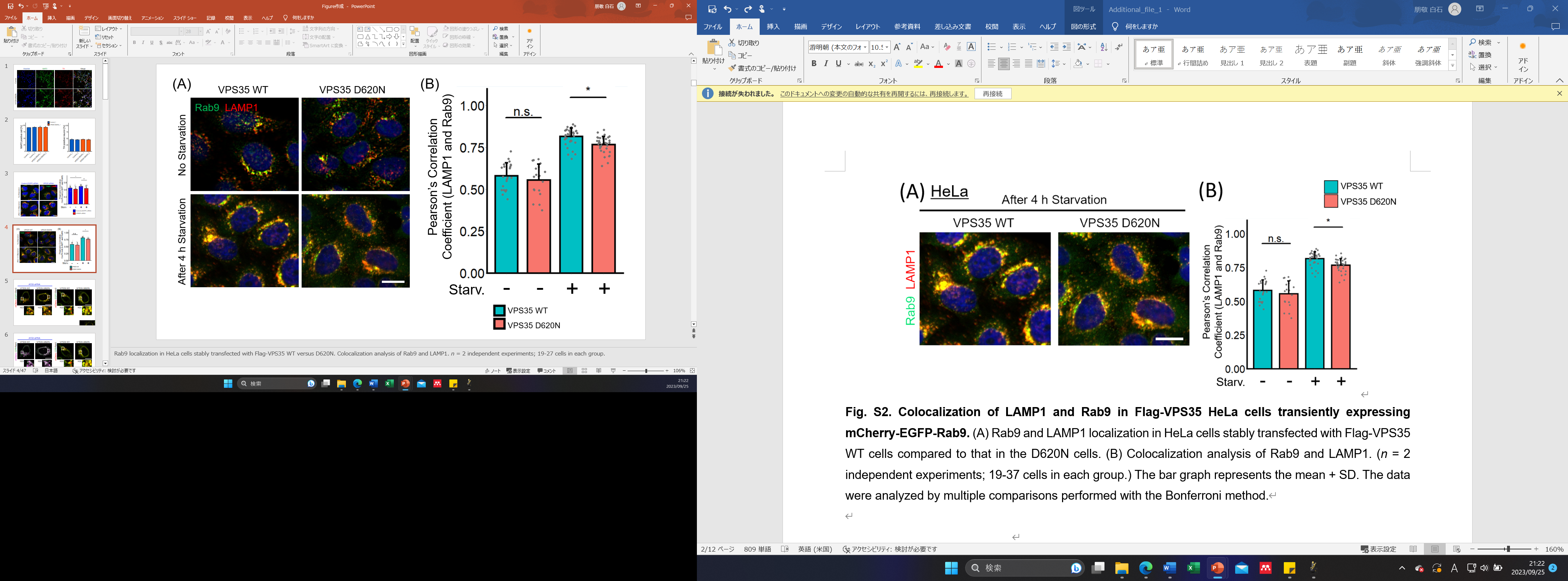


**Fig. S2 Colocalization of LAMP1 and Rab9 in Flag-VPS35 HeLa cells transiently expressing mCherry-EGFP-Rab9.** (A) Rab9 and LAMP1 localization in HeLa cells stably transfected with Flag-VPS35 WT cells compared to that in the D620N cells. (B) Colocalization analysis of Rab9 and LAMP1. (*n* = 2 independent experiments; 19-37 cells in each group.) The bar graph represents the mean + SD. The data were analyzed by multiple comparisons performed with the Bonferroni method. Scale bar = 10 μm


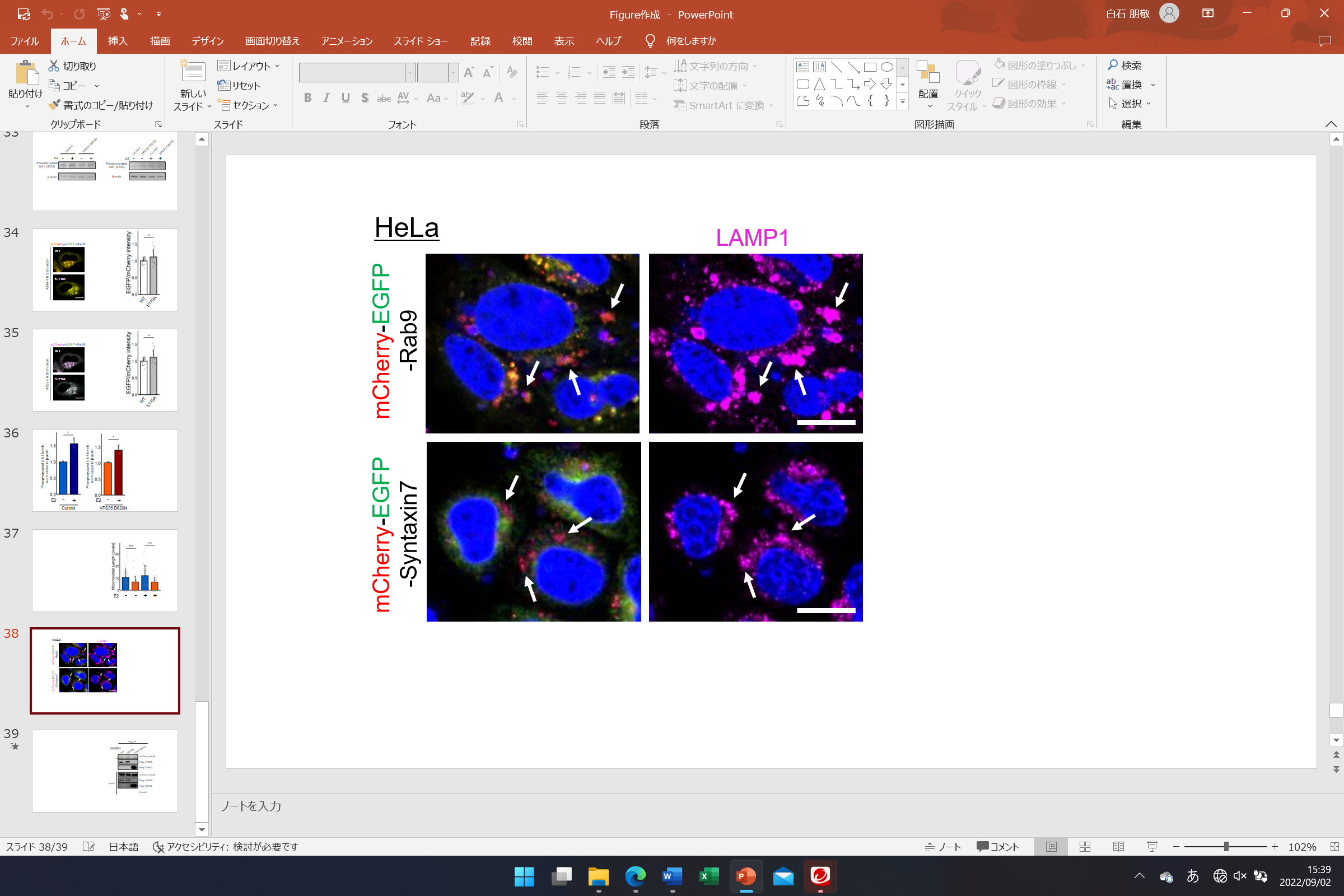


**Fig. S3 Colocalization of LAMP1 and red puncta in Flag-VPS35 HeLa cells transiently expressing mCherry-EGFP-Rab9 or mCherry-EGFP-Syntaxin7 protein.** Red puncta of mCherry-EGFP-Rab9/Syntaxin7 proteins were colocalized with LAMP1 positive organelles. Scale bar = 10 μm


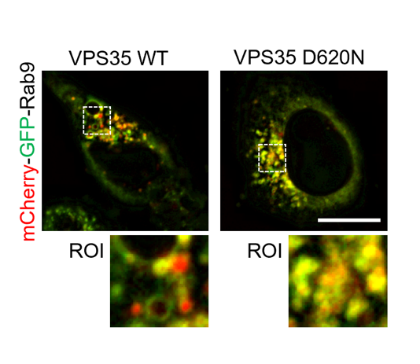


**Fig. S4 Representative images showing Flag-VPS35 HeLa cells transiently expressing mCherry-EGFP-Rab9 protein.** Red puncta indicate mCherry-EGFP-Rab9 proteins in acidic organelles. Scale bar = 10 μm


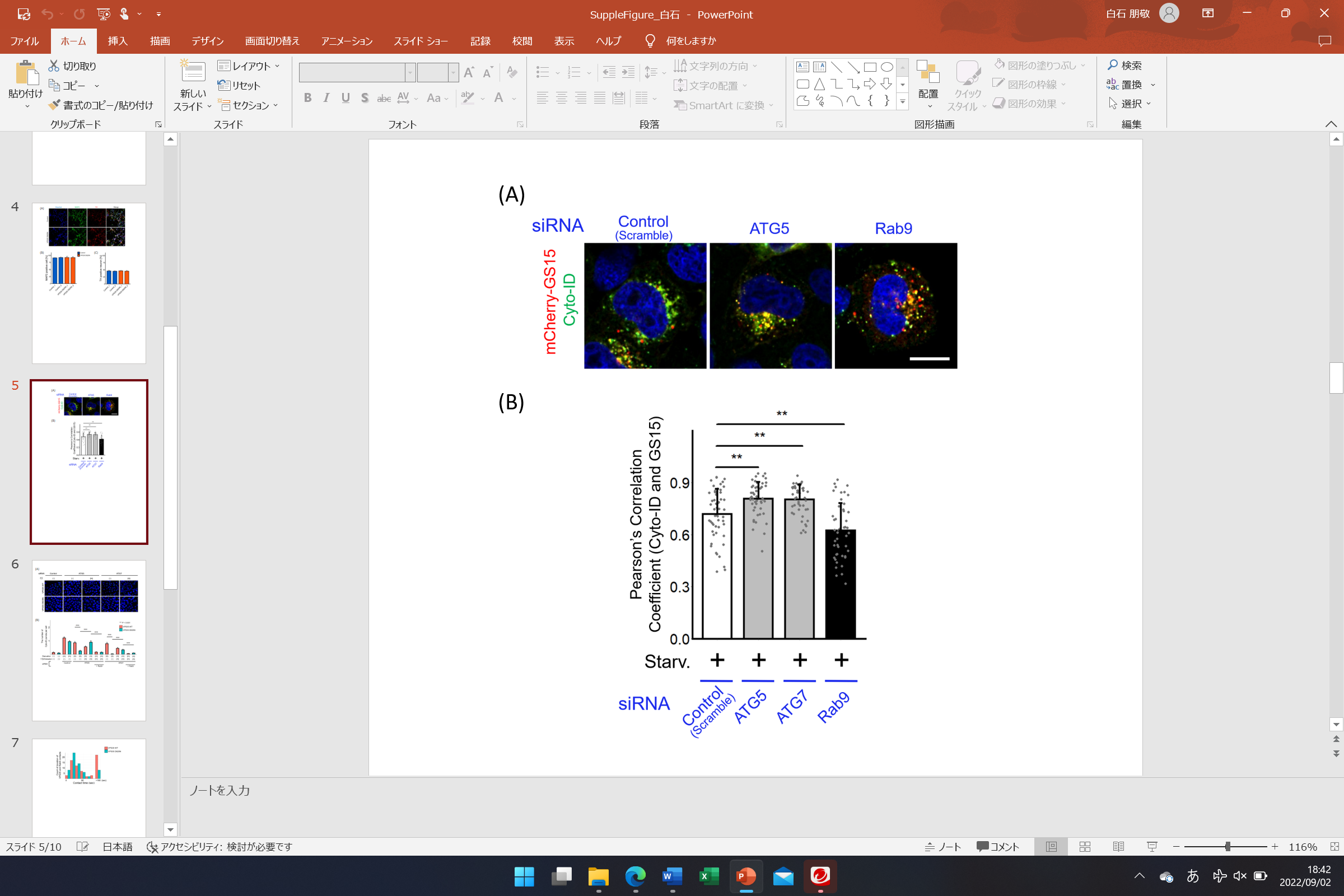

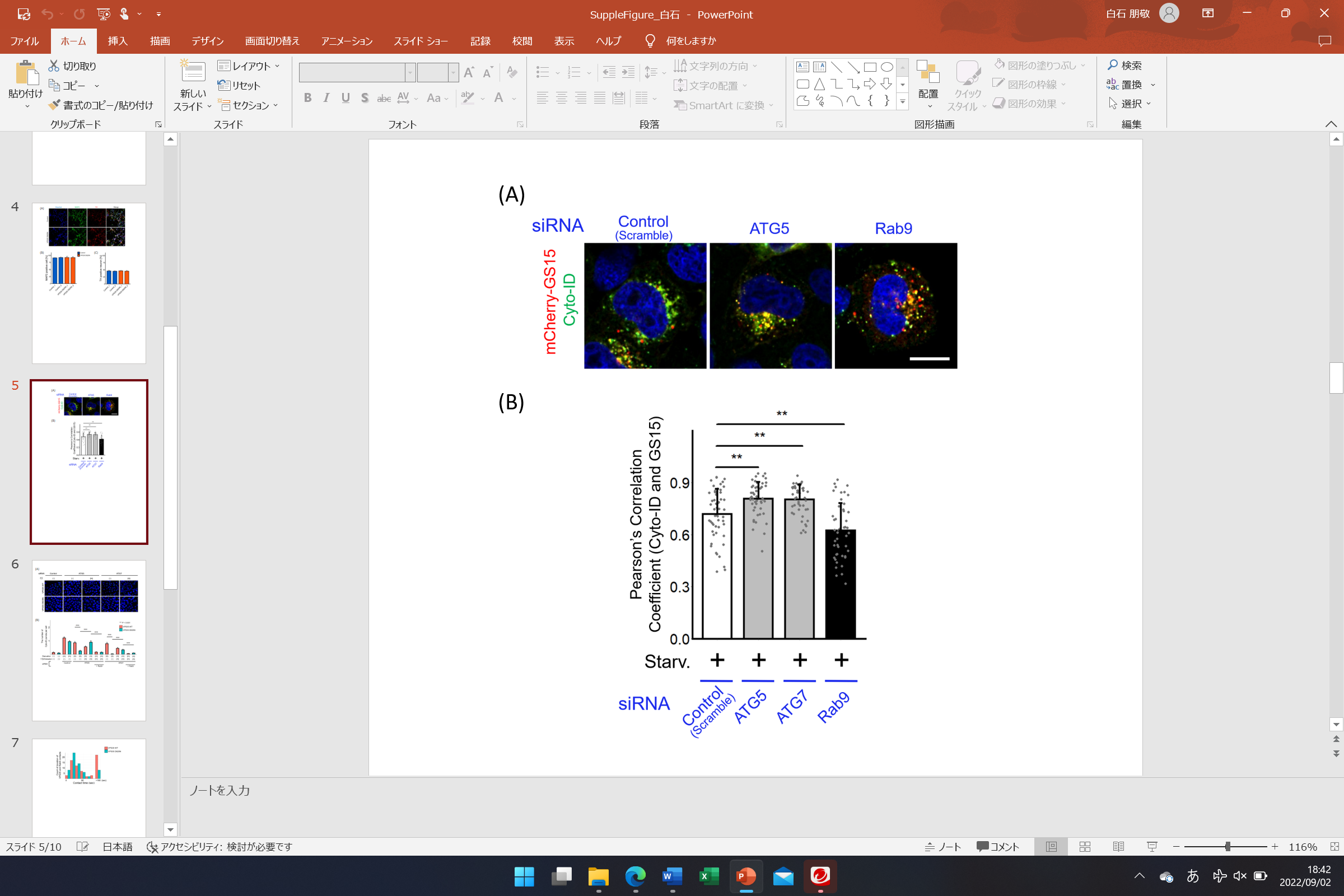

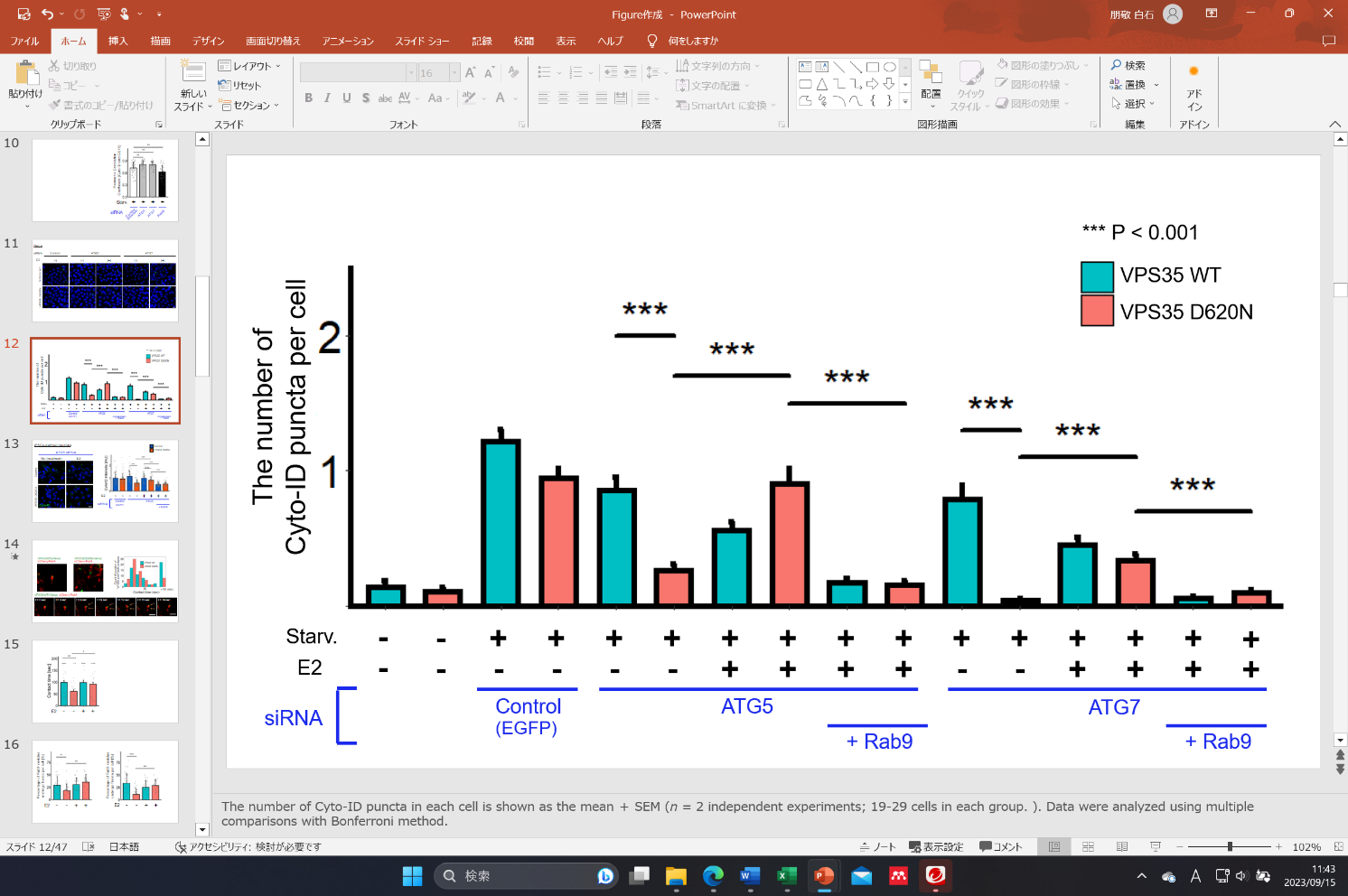

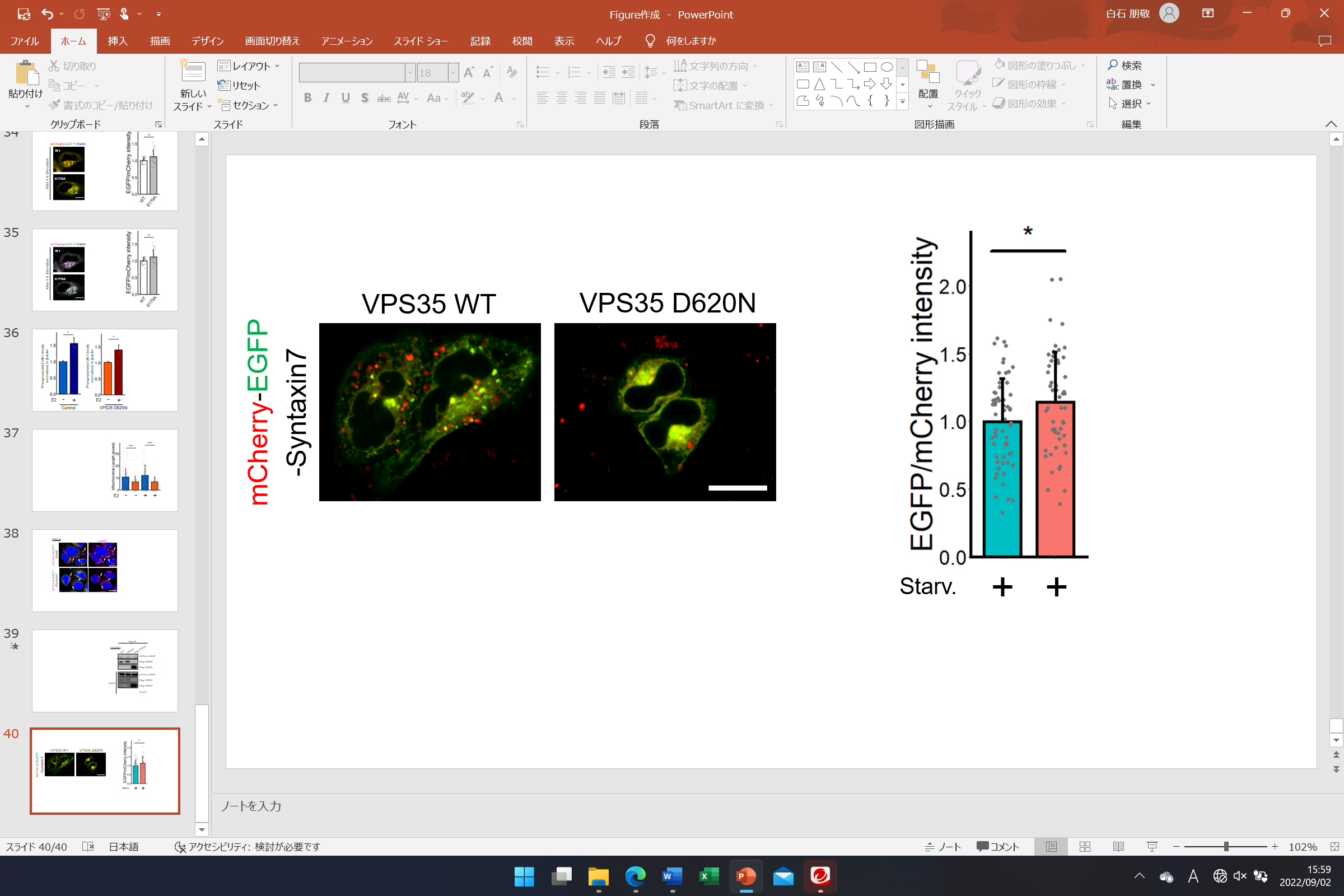


**Fig. S5 The effect of the VPS35 D620N mutation on lysosomal localization of syntaxin7.** (A) Representative images showing Flag-VPS35 HeLa cells transiently expressing mCherry-EGFP-Syntaxin7 protein. Red signals indicate mCherry-EGFP-Syntaxin7 proteins in acidic organelles. Scale bar = 10 μm. (B) The difference in the signal intensity ratio of EGFP/mCherry in Flag-VPS35 WT and D620N HeLa cells transiently expressing the mCherry-EGFP- Syntaxin7 protein. Three independent experiments were performed. The bar graph represents the mean + SD. The data were analyzed by Student’s *t* test


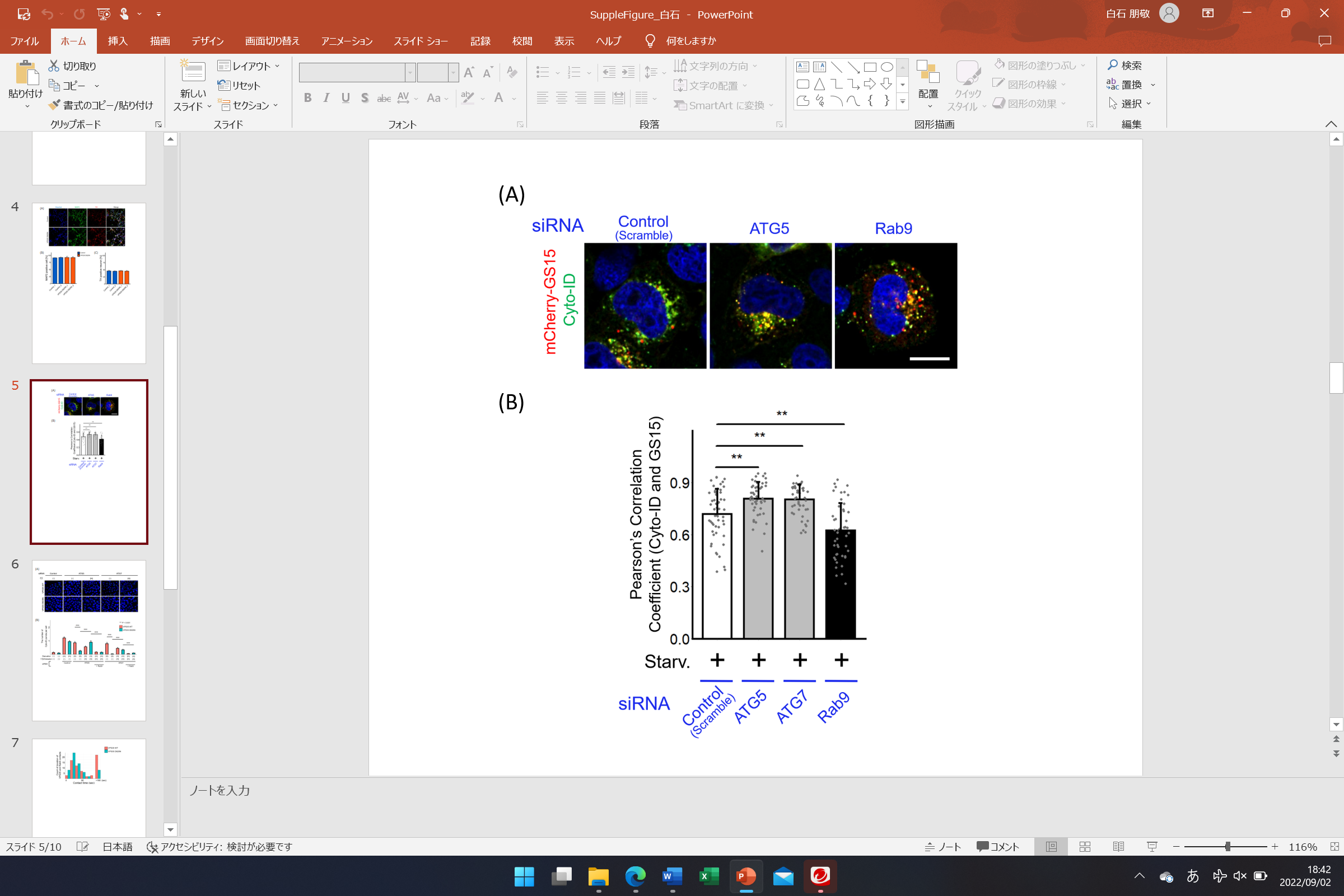


**Fig. S6** **Analysis of colocalization of GS15 and Cyto-ID puncta after conventional or alternative autophagy suppression**. (A) Autophagic vacuoles in Flag-VPS35 HeLa cells transiently transfected with mCherry-GS15 were analyzed by Cyto-ID. Colocalization analysis of Cyto-ID and mCherry-GS15. (*n* = 3 independent experiments; 47-54 cells in each group.) The bar graph represents the mean + SD. The data were analyzed by multiple comparisons performed with the Bonferroni method. Scale bar = 10 μm


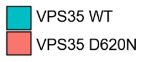
**
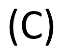
**
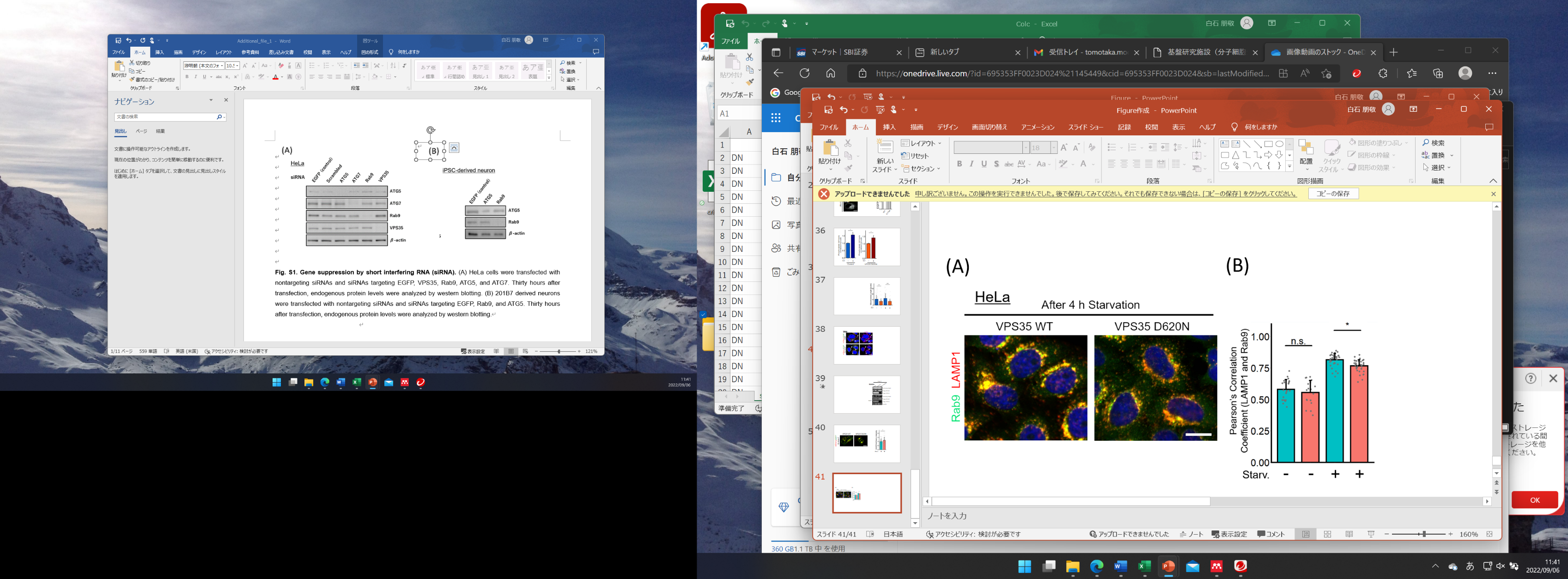

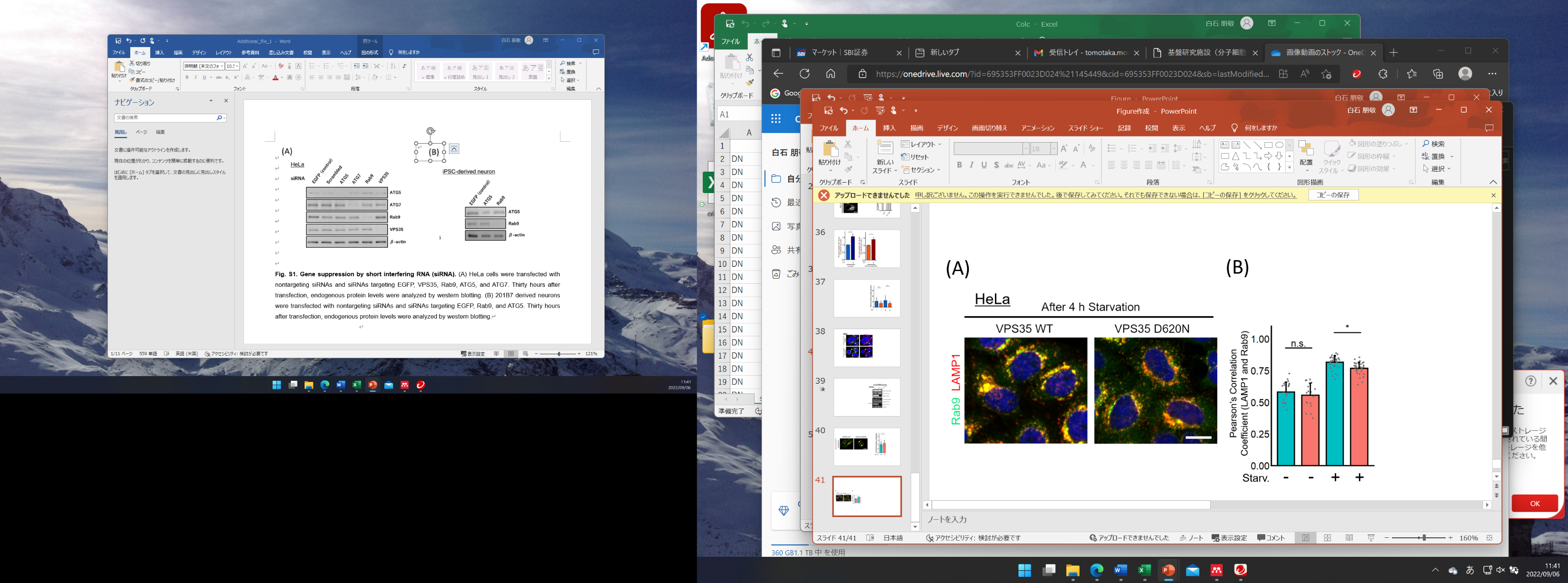


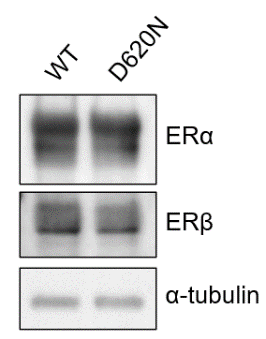

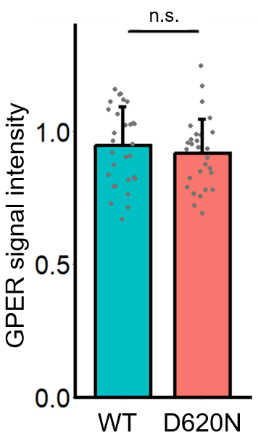


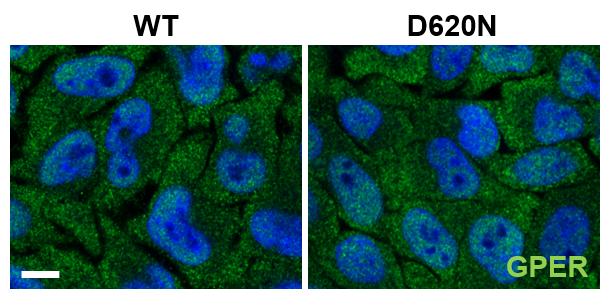


**Fig. S7 Comparison of estrogen receptors between VPS35 WT and D620N cells**. (A) The lysates of HeLa cells stably transfected with VPS35 WT or D620N were analyzed by SDS–PAGE. (B) HeLa cells stably transfected with VPS35 WT or D620N were stained with GPER. (C) The signal intensities of GPER in each cell were plotted. (*n* = 3 independent experiments; 30 cells in each group.) The bar graph represents the mean + SD. ER, estrogen receptor; GPER, G-protein-coupled estrogen receptor. Scale bar = 10 μm


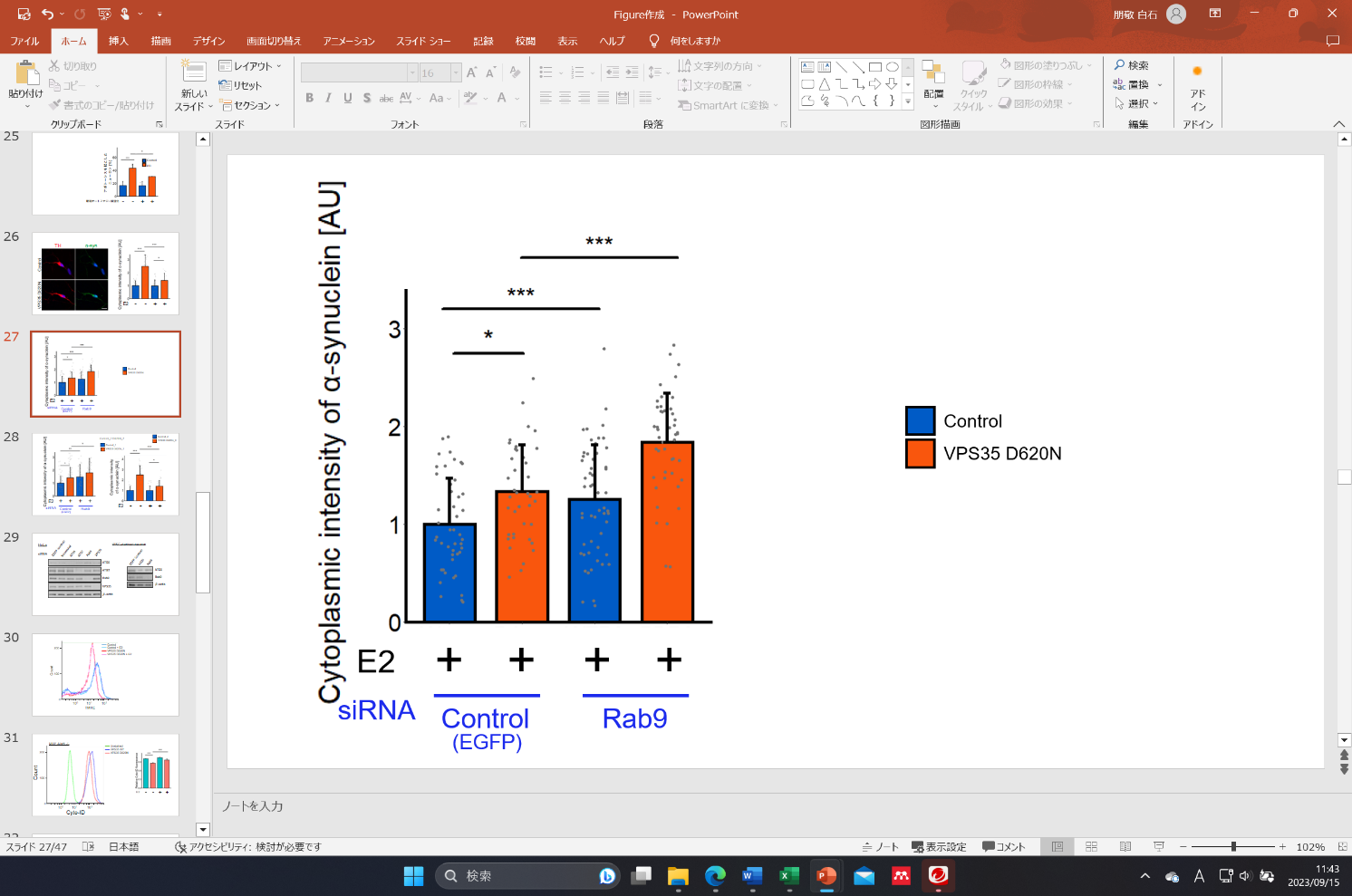

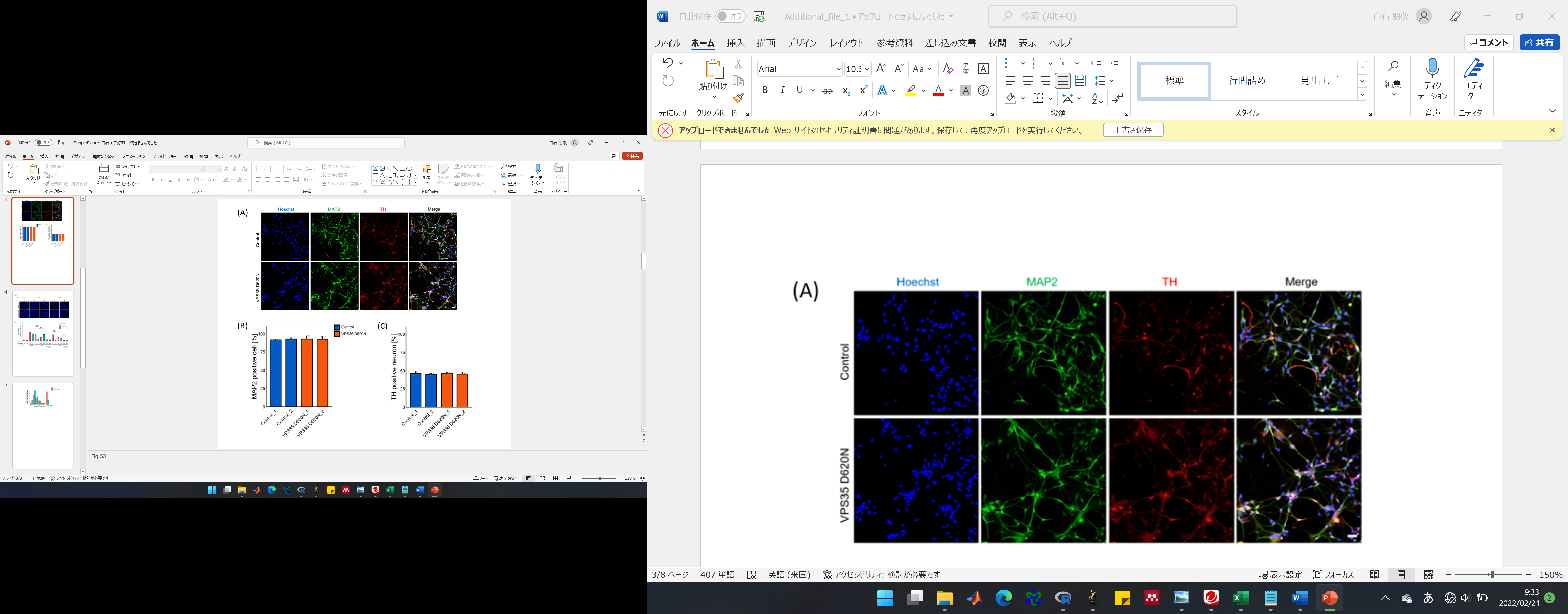


**Fig. S8 Differentiation and characterization of iPSC-derived dopaminergic neurons.** (A) Immunostaining of differentiated dopaminergic neurons. MAP2 (green, neuronal marker) and TH (red, dopaminergic neuron marker) are shown in the control and VPS35 D620N cells. Scale bar = 20 μm. (B, C) Frequency of MAP2-positive cells or TH-positive neurons in a single field. (n = 3, independent experiments.) The bar graph represents the mean + SD


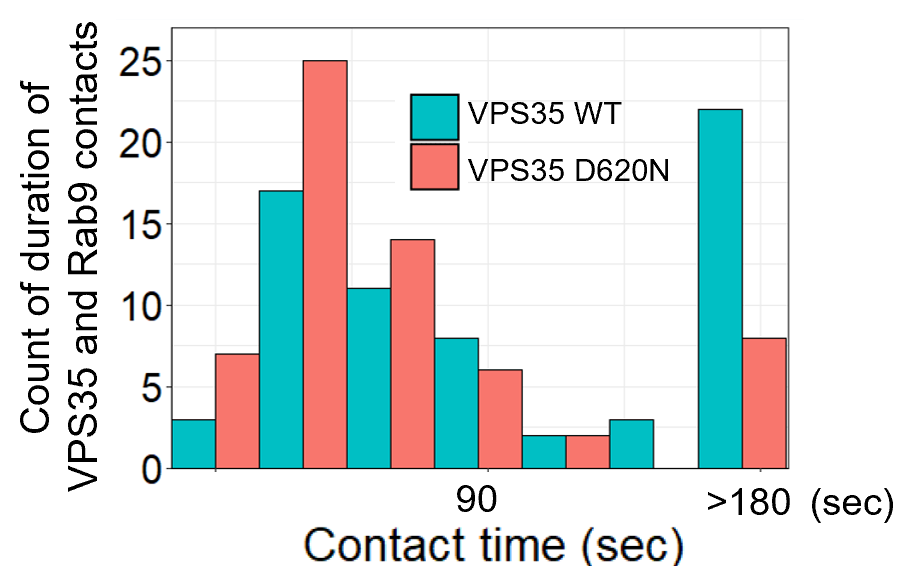


**Fig. S9** **Histogram showing the duration of VPS35 and Rab9 contact.** HeLa cells were transiently transfected with VPS35-Venus and mCherry-Rab9. All VPS35 and Rab9 contacts established before the beginning of the video were analyzed. Contacts that lasted throughout the entire 3-min video were categorized as >180 sec in the histogram

#
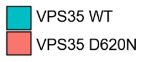

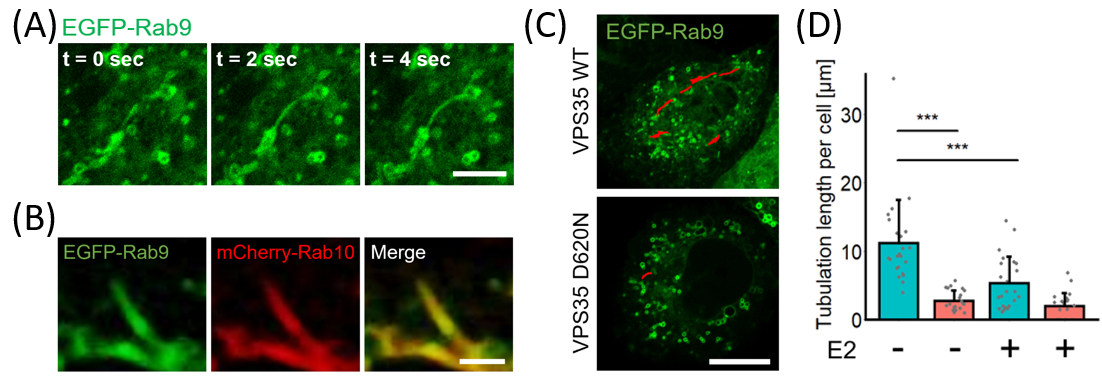


**Fig. S10 Analysis of Rab9 endosome tubulation.** (A) Live-cell imaging in HeLa cells transiently expressing EGFP-Rab9 showing tubule formation with Rab9 vesicles. Scale bar = 2 μm. (B) Live-cell imaging showing EGFP-Rab9 and mCherry-Rab10 in HeLa cells. Scale bar = 1 μm. (C) Trajectories (red lines) of the tubule formation with Rab9 vesicles in the 3-min videos. (D) Comparison of total tubule length in each cell. (n = 2 independent experiments; 21-23 cells in each group.) All data were analyzed using Kruskal-Wallis test, followed by multiple comparisons through the Bonferroni method. * p < 0.05, ** p < 0.01, and *** p < 0.001. The bar graph represents the mean + SD

**
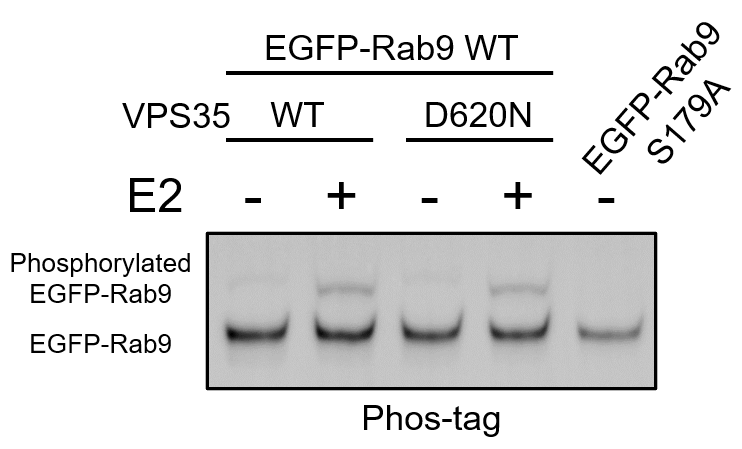
**

**Fig. S11 Rab9 phosphorylation by estrogen in HeLa cells stably transfected with Flag-VPS35 WT or D620N.** VPS35 WT or D620N HeLa cells transiently expressing EGFP-Rab9 WT or S179A were treated with estrogen. The lysate was analyzed by SDS–PAGE with Phos-tag.


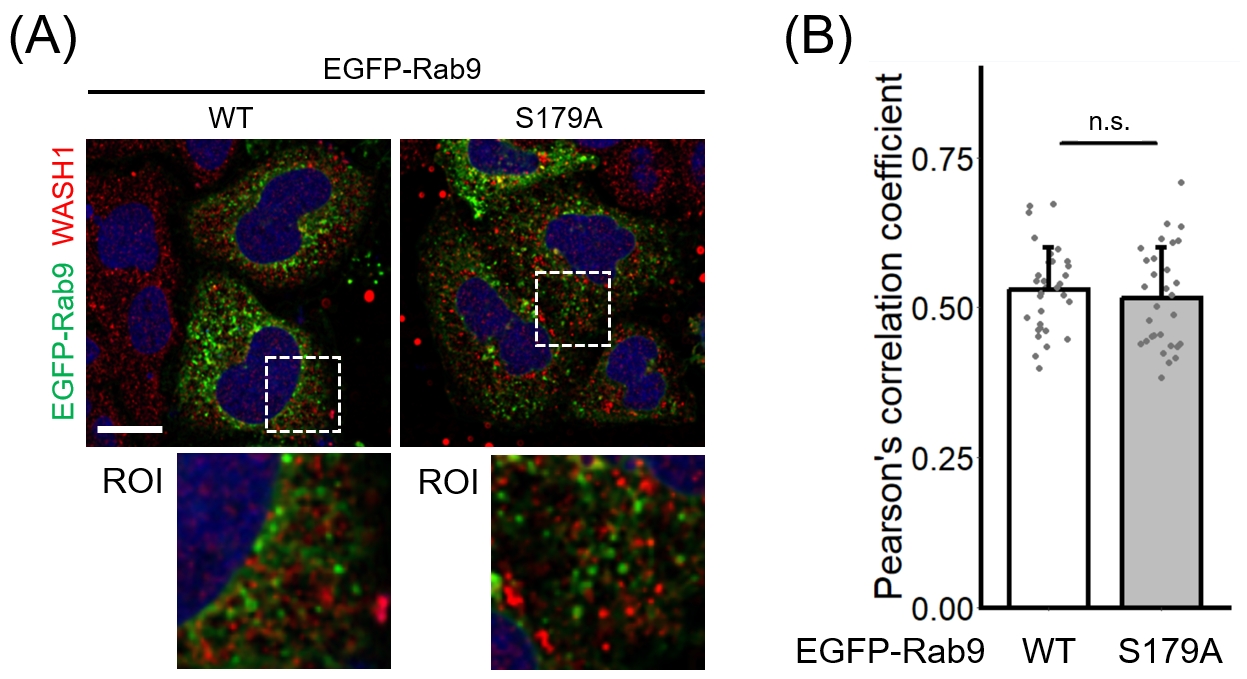


**Fig. S12 Colocalization analysis of Rab9 and WASH1 in HeLa cells transiently transfected with EGFP-Rab9 WT or S179A.** (A) EGFP-Rab9 and WASH1 localization in HeLa cells transiently transfected with EGFP-Rab9 WT or S179A. (B) Colocalization analysis of EGFP-Rab9 and WASH1. (*n* = 3 independent experiments; 30 cells in each group.) Data were analyzed using *t*-test. The bar graph represents the mean + SD. Scale bar = 10 μm


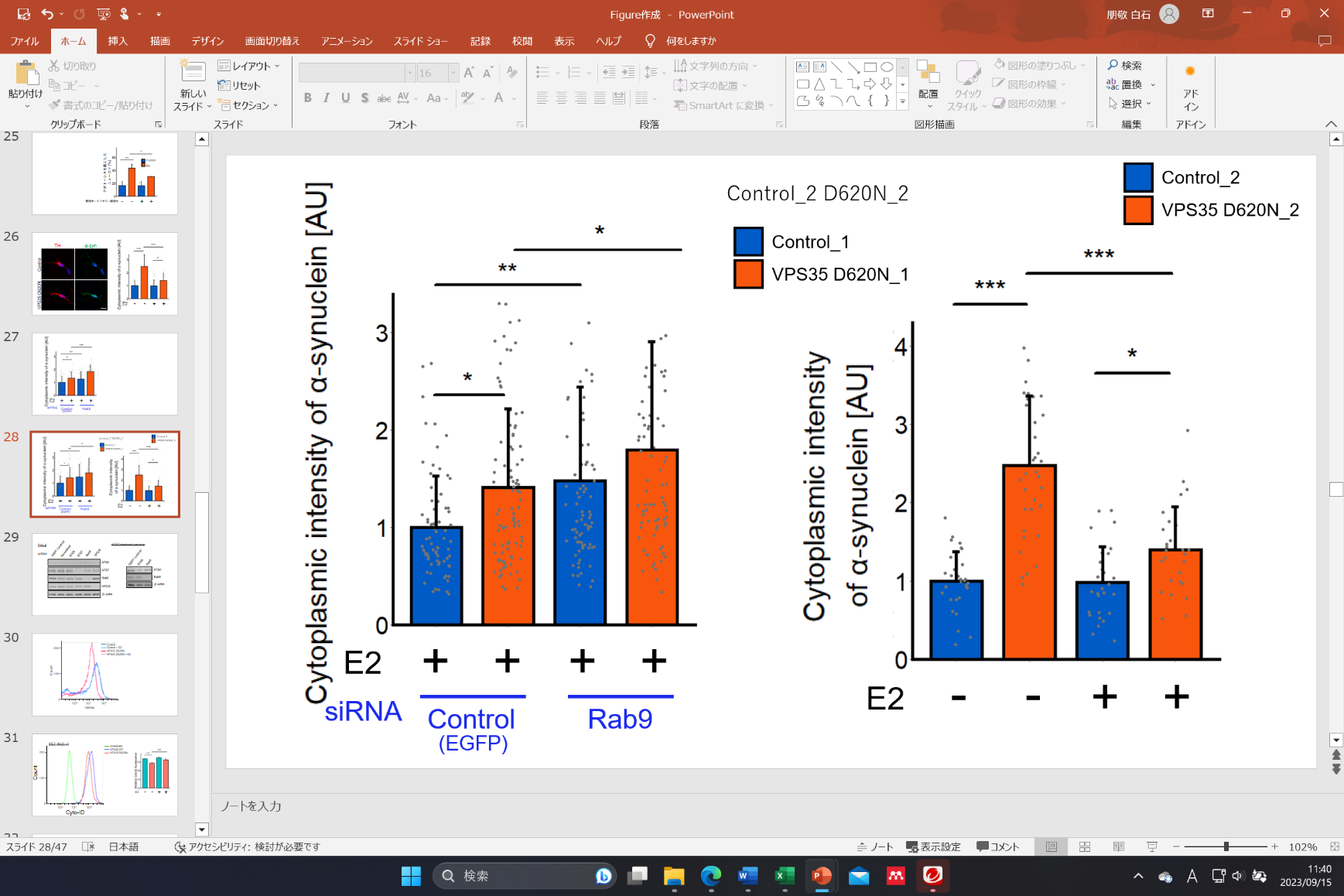

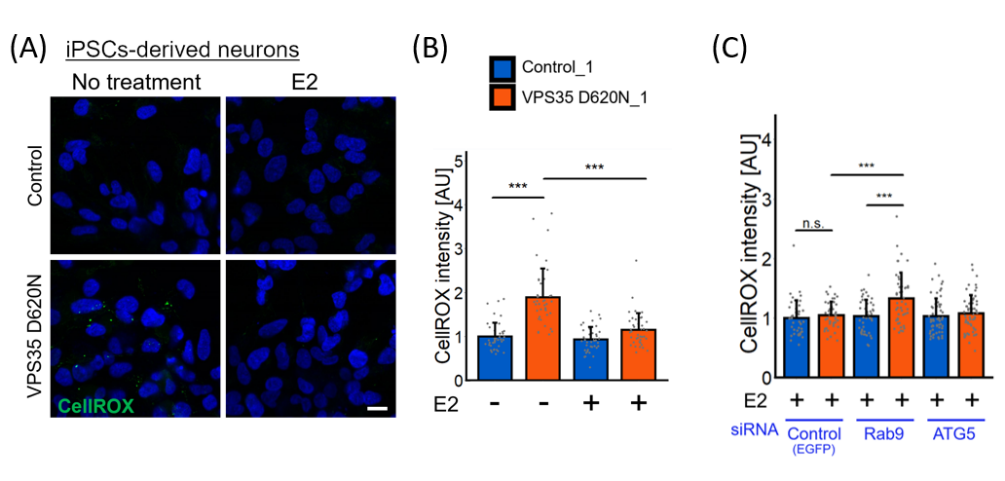


**Fig. S13** **The antioxidative effect of estrogen depends on Rab9 expression in patient-derived VPS35 D620N neurons.** (A) Results of an oxidative stress analysis using CellROX green reagent in differentiated dopaminergic neurons derived from control and VPS35 D620N iPSCs. Scale bar = 10 µm. (B, C) Quantitation of CellROX intensity per cell area. (*n* = 2 independent experiments; 40-45 cells in each group.) The bar graph represents the means＋SD. The data were analyzed by multiple comparisons performed with the Bonferroni method.


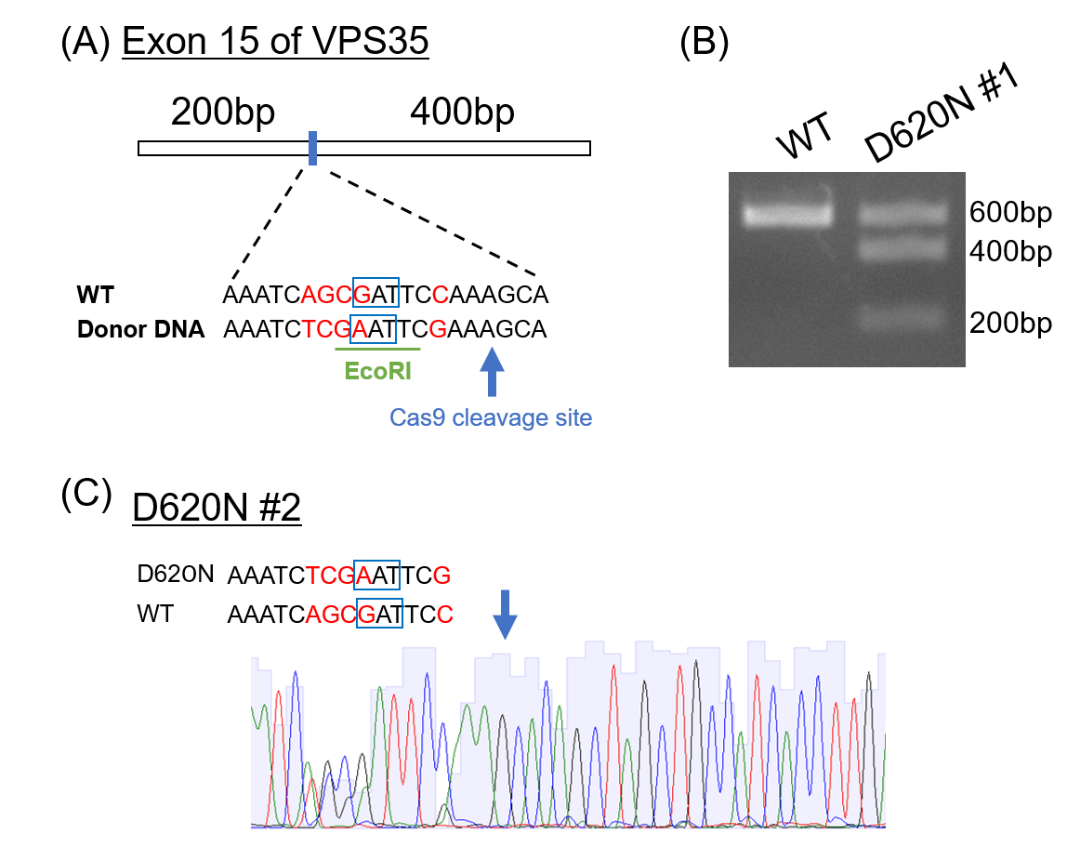


**Fig. S14 Generation of heterozygous VPS35 D620N SH-SY5Y cells.** (A) The method for introducing the D620N mutation into the VPS35 gene using CRISPR-Cas9 technology is illustrated in a diagram. The upper part of the diagram displays a portion of the exon15 of VPS35. The GAT sequence responsible for encoding the aspartic acid at position 620 is highlighted by blue lines. The lower part of the diagram illustrates the template used to create the D620N mutation. Additionally, there are silent substitutions in the sequence to establish an EcoRI restriction site, shown in green. (B) Restriction fragment length polymorphism analysis using the EcoRI enzyme on a 604-bp genomic DNA region including the variant. (C) Through Sanger sequencing, the presence of the D620N mutation in one of the two VPS35 alleles was confirmed. The sequencing outcome for D620N #1 is depicted in Fig.5A


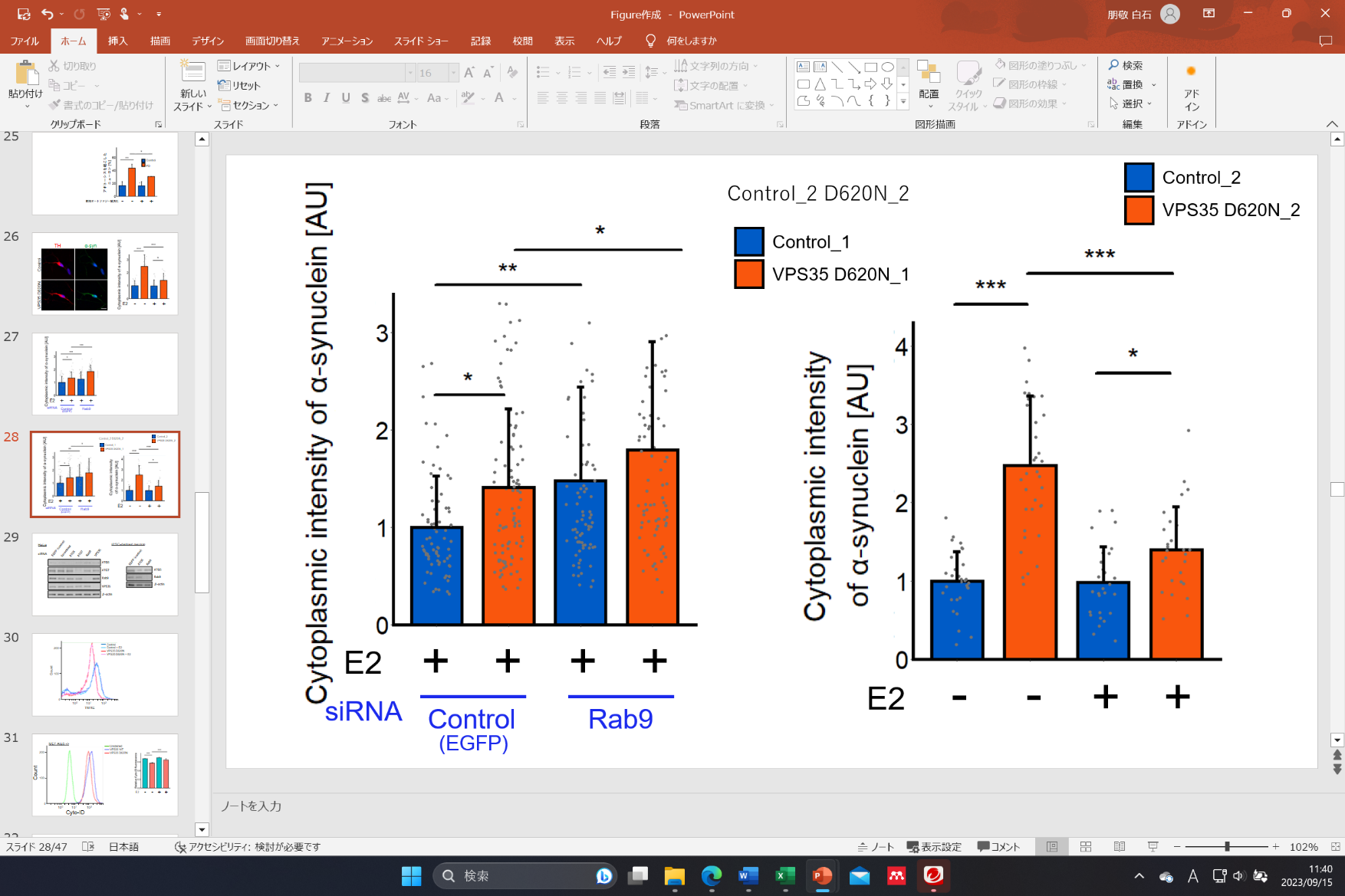

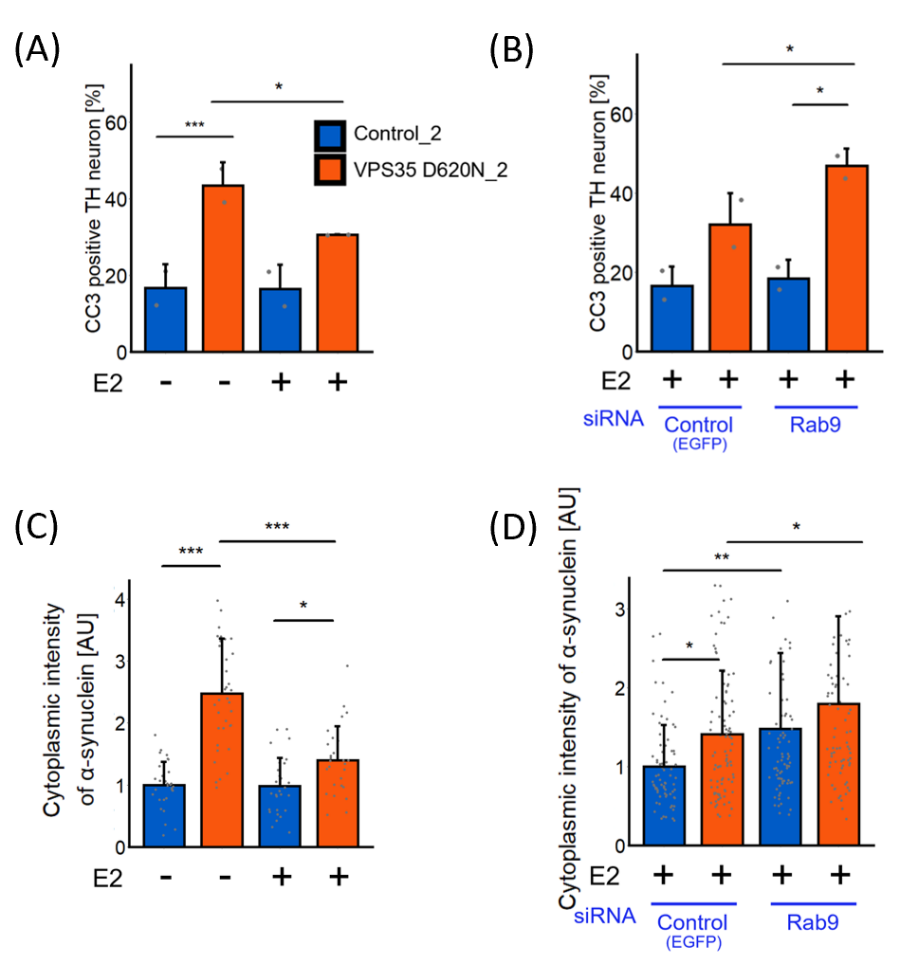


**Fig. S15** **The neuroprotective effect of estrogen depends on Rab9 in patient-derived VPS35 D620N neurons.** The experiments described in Fig. 5 were also confirmed with Control_2 and VPS35 D620N_2 cells. (A, B) Frequencies of CC3-positive neurons in each group. The data were analyzed by chi-square. (n = 2 independent experiments; 109-131 cells in each group.) (C, D) Quantitation of the cytoplasmic intensity of α-synuclein. The data were analyzed by multiple comparisons performed with the Bonferroni method. (n = 2 independent experiments; 74-90 cells in each group.) The bar graph represents the means+SD

**
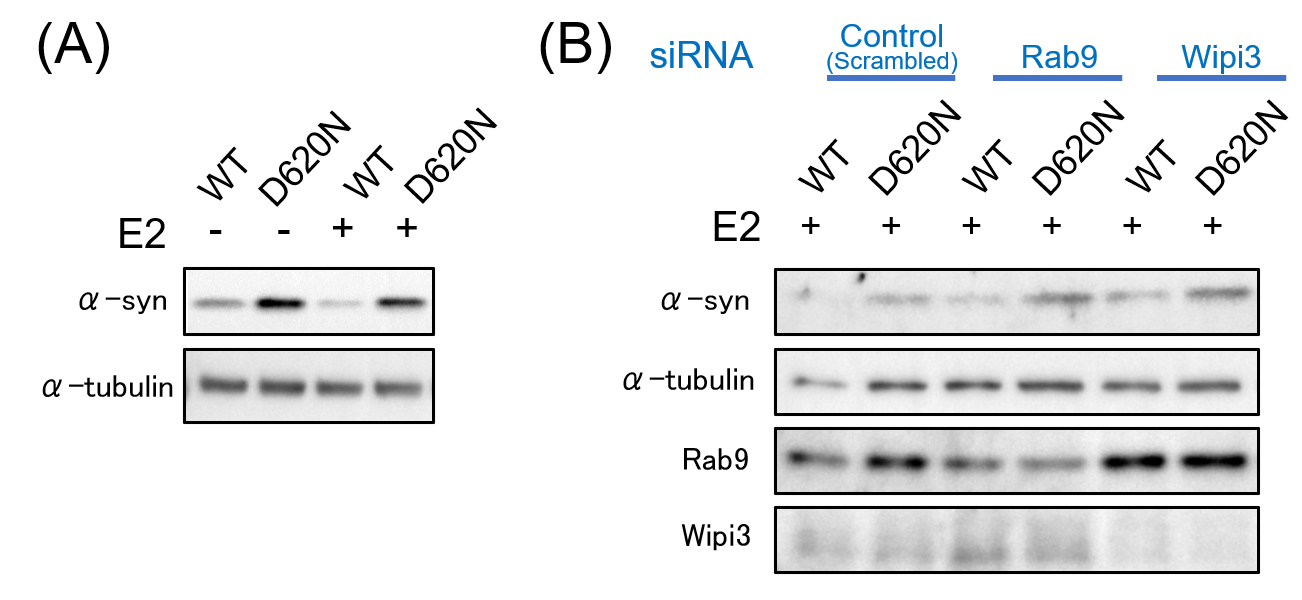
**

**Fig. S16 Comparison of α-synuclein levels with or without estrogen treatment in the differentiated WT or D620N #2 SH-SY5Y cells.** The experiments described in Fig. 6J and K were also confirmed with WT or D620N #2 SH-SY5Y cells. (A) The lysates of CRISPR/Cas9 generated SH-SY5Y WT or D620N cells were analyzed by SDS–PAGE. (B) Scrambled, Rab9, or Wipi3 siRNA were transfected in the cells shown in (B).
