# Supplementary material for: The impact of VPS35 D620N mutation on alternative autophagy and its reversal by estrogen in Parkinson's disease
Source: Cell Mol Life Sci. 2024 Feb 27;81(1):103. doi: 10.1007/s00018-024-05123-4 (PMC10896810; doi:10.1007/s00018-024-05123-4)
Supplement: Supplementary file 2 — Supplementary file2 (DOCX 14 KB) [file 18_2024_5123_MOESM2_ESM.docx]

**Additional file 2:**

**Supplementary Video S1** Live-cell imaging of VPS35(WT)-Venus and mCherry-Rab9

**Supplementary Video S2** Live-cell imaging of VPS35(D620N)-Venus and mCherry-Rab9

**Supplementary Video S3** Live-cell imaging of EGFP-Rab9 in a HeLa cell stably expressing Flag-VPS35 WT

**Supplementary Video S4** Live-cell imaging of EGFP-Rab9 in a HeLa cell stably expressing Flag-VPS35 D620N

**Additional file 3:**

**Supplementary Video S5** Live-cell imaging of EGFP-Rab9 and mCherry-Rab10

**Supplementary Video S6** Live-cell imaging of EGFP-Rab9

**Supplementary Video S7** Live-cell imaging of mCherry-Rab10

**Supplementary Video S8** Live-cell imaging of EGFP-Rab9 and mCherry-Rab10

**Supplementary Video S9** Live-cell imaging of EGFP-Rab9

**Supplementary Video S10** Live-cell imaging of mCherry-Rab10
