# Supplementary figures and images for: The impact of VPS35 D620N mutation on alternative autophagy and its reversal by estrogen in Parkinson's disease
Source: Cell Mol Life Sci. 2024 Feb 27;81(1):103. doi: 10.1007/s00018-024-05123-4 (PMC10896810; doi:10.1007/s00018-024-05123-4)

## Slide 1
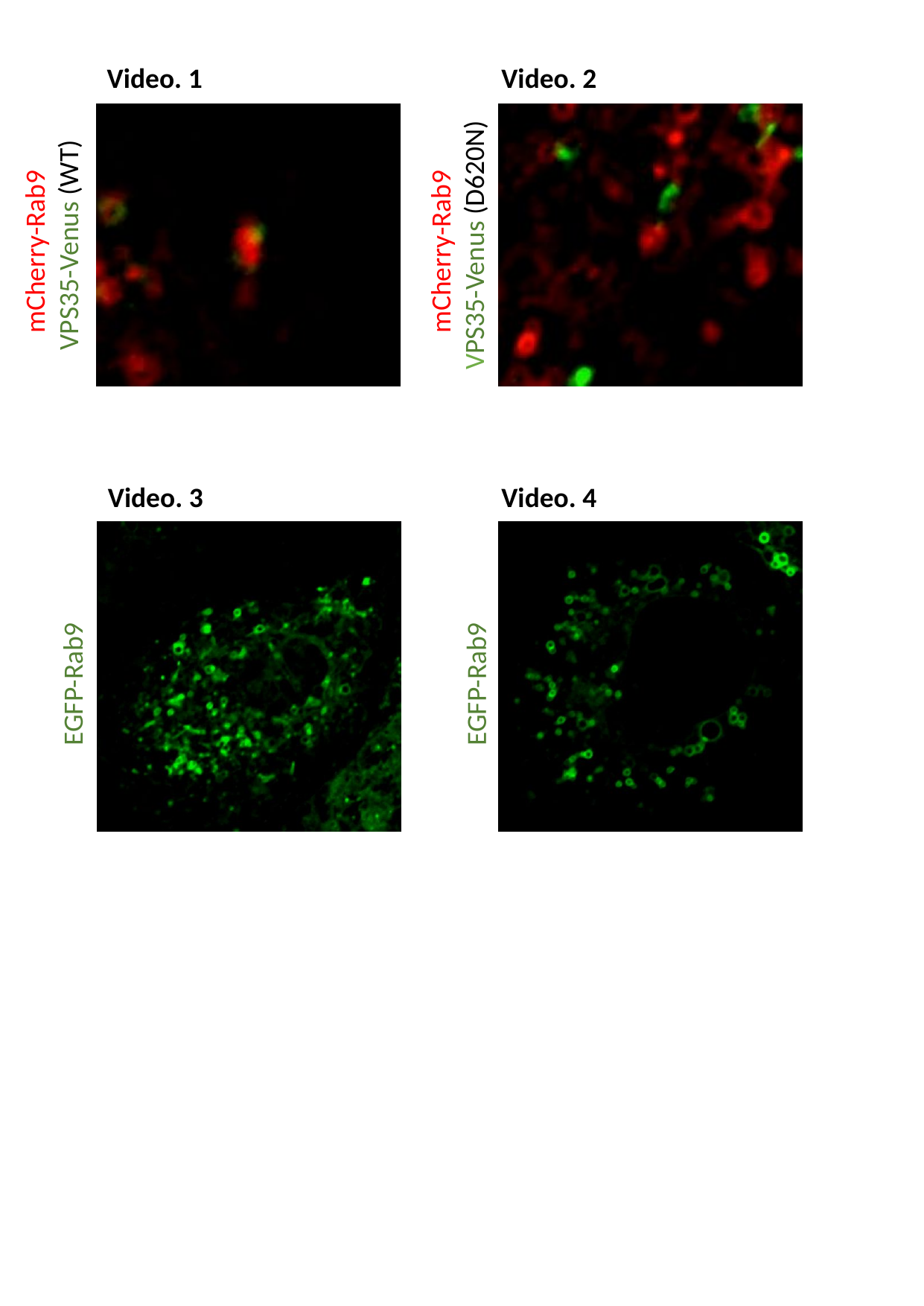

Video. 1
Video. 2
mCherry-Rab9
VPS35-Venus (D620N)
mCherry-Rab9
VPS35-Venus (WT)
Video. 3
Video. 4
EGFP-Rab9
EGFP-Rab9

Supplement: Supplementary file 3 — Supplementary file3 (PPTX 16012 KB) [file 18_2024_5123_MOESM3_ESM.pptx]
