# Supplementary material for: The impact of VPS35 D620N mutation on alternative autophagy and its reversal by estrogen in Parkinson's disease
Source: Cell Mol Life Sci. 2024 Feb 27;81(1):103. doi: 10.1007/s00018-024-05123-4 (PMC10896810; doi:10.1007/s00018-024-05123-4)
Supplement: Supplementary file 4 — Supplementary file4 (PPTX 6079 KB) [file 18_2024_5123_MOESM4_ESM.pptx]

## Slide 1
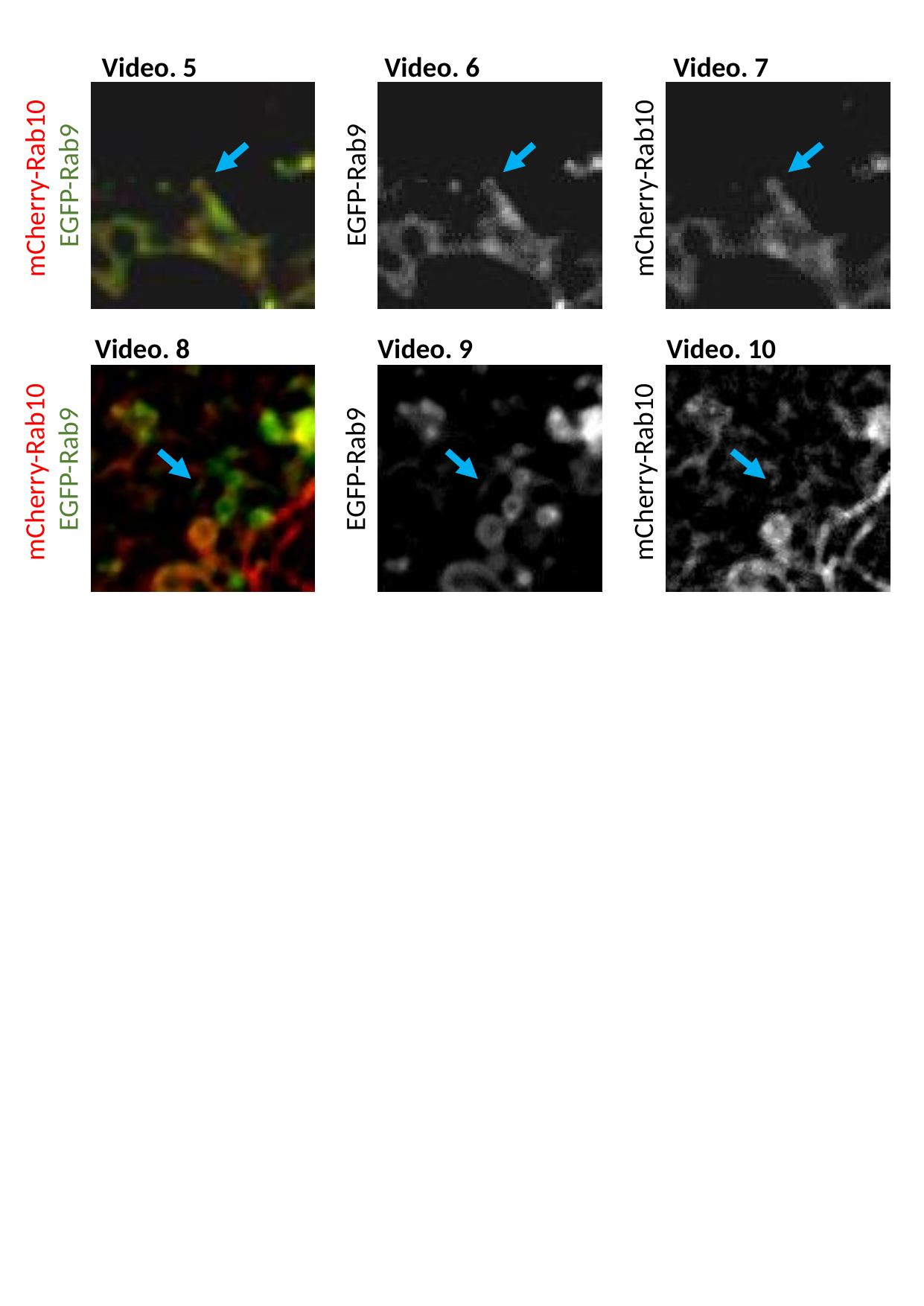

Video. 5
Video. 6
Video. 7
mCherry-Rab10
EGFP-Rab9
EGFP-Rab9
mCherry-Rab10
Video. 8
Video. 9
Video. 10
mCherry-Rab10
EGFP-Rab9
EGFP-Rab9
mCherry-Rab10
